# Integrated Spatial Simulation of Population and Urban Land Use: a Pan-European Model Validation

**DOI:** 10.1007/s12061-023-09518-x

**Published:** 2023-06-01

**Authors:** Vasco Diogo, Chris Jacobs-Crisioni, Claudia Baranzelli, Carlo Lavalle

**Affiliations:** 1 Swiss Federal Research Institute WSL, Land Change Science Unit, Zuercherstrasse 111, CH-8903 Birmensdorf, Switzerland; 2 European Commission, Joint Research Centre, Directorate B - Growth and Innovation, Territorial Development Unit (B3), Via Enrico Fermi 2749, Ispra, VA 21027 Italy

**Keywords:** Spatial modelling, Residential land use, Population, Degree of urbanisation, Validation, European Union

## Abstract

Spatial models jointly simulating population and land-use change provide support for policy-making, by allowing to explore territorial developments under alternative scenarios and resulting impacts in the environment, economy and society. However, their ability to reproduce observed spatial patterns is rarely evaluated through model validation. This lack of insight prevents researchers and policy-makers of fully grasping the ability of existing models to provide sensible projections of future land use and population density. In this article, we address this gap by performing a model validation of the LUISA Territorial Modelling Platform, a spatial model jointly simulating population and land use at a fine resolution (100 m) in the European Union and United Kingdom. In particular, we compare observed and simulated patterns of population and urban residential land-use change for the period of 1990–2015, and evaluate the model performance according to different degrees of urbanisation. The results show that model performance can vary depending on the context, even when the same data and methods are uniformly applied. The model performed consistently well in urban areas characterized by compact urban growth, but poorly where residential development occurred predominantly in scattered patterns across rural areas. Overall, the model tends to favour the formation of densely populated, highly accessible urban conglomerations, which often do not entirely correspond to the observed patterns. Based on the validation results, we propose directions for further model improvement and development. Model validation should be regarded as a critical step, and an integral part, in the process of developing models for policy support.

**Supplementary Information:**

The online version contains supplementary material available at 10.1007/s12061-023-09518-x.

## Introduction

Human land use and population are both crucial elements for assessing the supply and demand of ecosystem services, quality of life, vulnerability and adaptive capacity to (climate) risks (Chaplin-Kramer et al., 2019; Lavalle et al., 2017; Spake et al., 2017). Therefore, it is critical to have a good understanding of the potential drivers and trade-offs of future land-use trajectories and related changes in population patterns** (**Rounsevell et al., 2012). Spatial models of land-use change can be used as learning tools to test hypotheses and formalise knowledge on the functioning of land systems, enabling researchers and decision-makers to explore the complex interplay among multiple land-use drivers (Verburg et al., 2019). Such models also allow to investigate potential future developments and resulting impacts in the environment, economy and society, thus providing important insights to inform the elaboration of governance strategies for mitigating impacts and capitalising on opportunities in land systems (Lavalle et al., 2020).

The spatial patterns of human land use are intrinsically intertwined with those of population density (Mulder, 2008). For example, increases in population within a region contribute to increases in the demand for residential land use. In turn, an increase in residential land use usually promotes an increase in population density. Land use and population patterns are, however, usually modelled separately, by considering each other as an exogenous factor, even though they are simultaneously a driver and an outcome of one another (Rounsevell et al., 2014; Van Vliet et al., 2019). The following modelling approaches constitute the only few exceptions in which both population and land-use change patterns are jointly simulated in large-scale land-use models. Terama et al. (2017) and Zhou et al. (2019), for example, focused on simulating only urban land use and population patterns, thus not allowing to assess the potential trade-offs and interactions with other types of land use (e.g., agriculture, forestry). Van Vliet et al. (2012), White et al. (2012), and Baranzelli et al. (2014) proposed modelling approaches allowing for the simulation of multiple land-use sectors and population. In these approaches, urban expansion is simulated according to expert-based population threshold rules, through a chain of separate discrete allocation mechanisms Jacobs-Crisioni et al. (2017) presented an approach for simulating multiple land uses and population in a more integrated way, according to which, for each time-step, 1) projected changes in regional population are firstly translated into a measure of local demand for residential land use; 2) this local demand measure is then used as one of the drivers for the allocation of (residential) land use; and finally, 3) local population is allocated over the simulated residential land-use patterns.

However, the ability of these models to reproduce observed spatial patterns and processes has so far never been investigated through model validation. This lack of insight prevents researchers and policy-makers to fully understand the ability of existing models to explain observed processes and the extent to which they provide sensible projections of future land-use change and population density.

In this article, we address these gaps by performing a model validation of the LUISA (Land-Use Integrated Sustainability Assessment) Territorial Modelling Platform (Lavalle et al., 2020). LUISA is a pan-European modelling application jointly simulating the spatial distribution of multi-sectoral land use and residential population by operationalising the modelling framework proposed in Jacobs-Crisioni et al. (2017). We compare observed historical patterns of urban residential land use and population distribution with those simulated by the LUISA model in the European Union countries and United Kingdom (EU27 + UK), for the period between 1990 and 2015. By performing the model validation, we aim at answering the following research questions:

- To what extent is LUISA able to jointly simulate and correctly reproduce the observed patterns of both residential land use and population change in EU27+UK during the period between 1990 and 2015?
- What patterns and processes of population and land change appear to be well captured by such model? Which processes may require an improved representation?
- To what extent does model performance depend on the context where the model is applied?

The remaining of the article is structured as follows. Firstly, we briefly describe the LUISA modelling platform (Section "The LUISA Model"), and particularly the specification of the model for the purpose of simulating multi-sectoral land use and population patterns in EU27 + UK during 1990–2015. Then, we describe the methods for performing the model validation (Section "Model Validation"). The results of the model validation are then presented in Section "Results", followed by a discussion and conclusions in Sections "Discussion" and "Conclusions", respectively.

## Material and Methods

### The LUISA Model

The LUISA Territorial Modelling Platform is a modelling application developed by the European Commission (EC) for jointly simulating multi-sectoral land use and residential population for all EU27 + UK countries at a fine resolution (100 m spatial grid). It has been designed to assess the direct and indirect impacts of EU policies with a territorial dimension (e.g. Trans-European Transport Network, Cohesion Policy and Common Agricultural Policy), in multiple sectors and across various geographical scales (Jacobs-Crisioni et al., 2017; Lavalle et al., 2020). LUISA makes use of an extensive knowledge base that includes EC’s reference demographic and macro-economic projections at the regional level (e.g. EC, 2016a, 2016b, 2015), and several thematic spatial datasets at detailed resolution (e.g. Donatelli et al., 2015; Florczyk et al., 2019; Rosina et al., 2020). These features allow LUISA to incorporate complex interactions between human activities and their context‐specific determinants, thus translating socioeconomic trends and policy scenarios into processes of territorial development (Perpiña Castillo et al., 2021; Proietti et al., 2022). The impacts of these developments are then quantified through the use of thematic indicators at various spatial aggregation levels, providing useful inputs for policy evaluation purposes in relation to relevant urban and rural development issues (Lavalle et al., 2017, 2020; Perpiña Castillo et al., 2018, 2021).

Five main groups of land-use types are currently modelled in LUISA: urban residential (i.e. built-up land used for residential functions), industry and commerce, agriculture, forest and (semi-)natural vegetation. Each country is simulated independently (except for Belgium and Luxembourg, which are modelled jointly) in 5-year time-steps, having land-use and population maps for a given reference year as a starting point. LUISA’s spatial outputs are maps representing population, land-use and accessibility patterns for each of the modelled time-steps (Jacobs-Crisioni et al., 2016, 2017; Lavalle et al., 2020). These outputs are then used to dynamically inform the starting point of the simulation in the next time step.

**Fig. 1:**
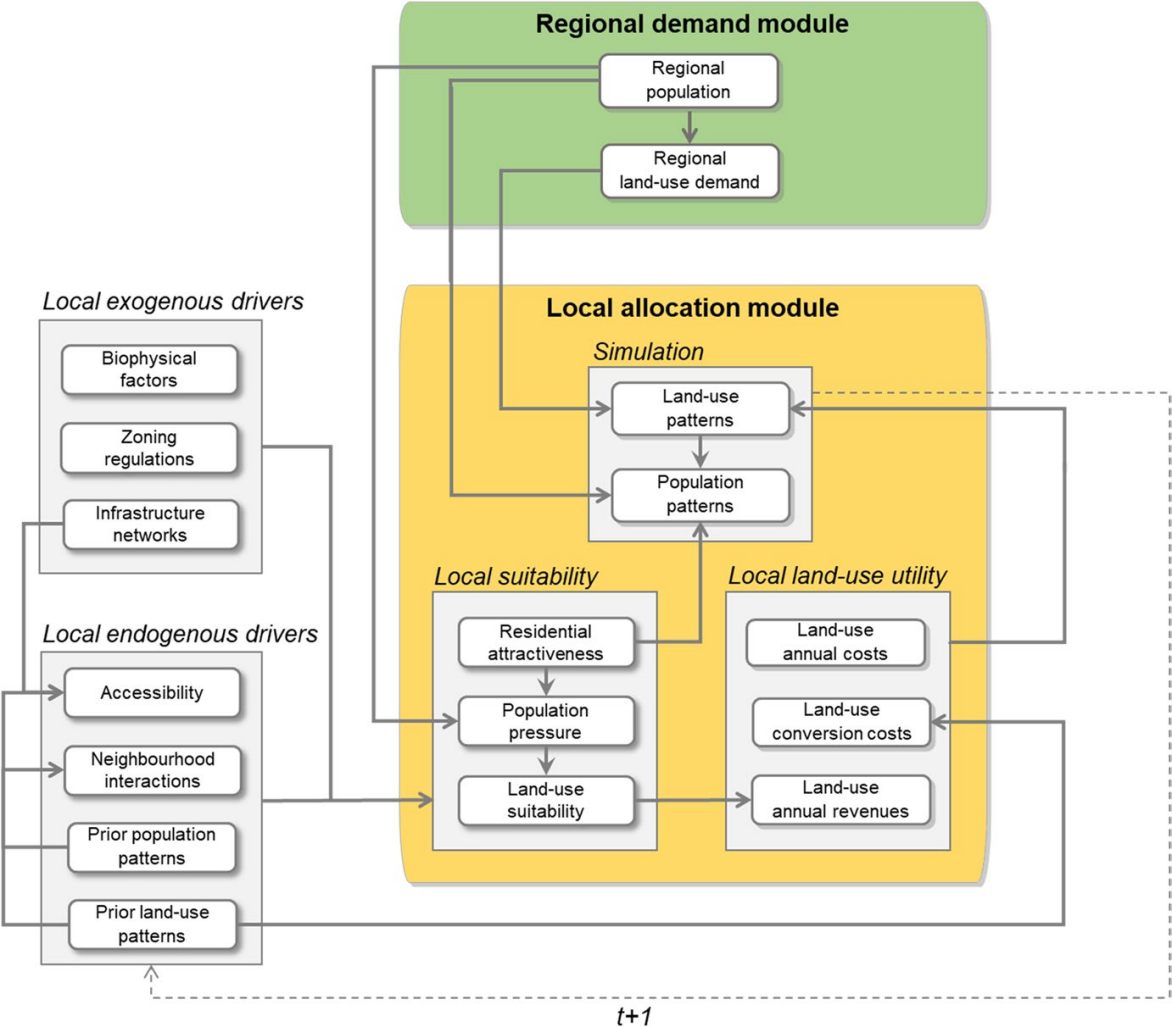
Modelling workflow of the LUISA Territorial Modelling Platform, including the regional demand and the local allocation modules

Developments in land systems result from complex interactions between multiple drivers operating across different scales (Hersperger et al., 2010; Plieninger et al., 2016). To capture these cross-scale interactions, LUISA’s model structure includes two interconnected modules: a regional demand module and a local allocation module (Fig. 1). In the regional demand module, the total regional population and land demand for different land-use activities are specified for each region, typically at NUTS^1^ 3 regional level and according to EC’s official projections and/or statistics (Batista e Silva et al., 2016). In the local allocation module, the model then allocates land use based on spatially-varying local land-use utilities and region-specific demands for competing land uses, and consecutively the local population distribution. Similarly to the utility-based framework for multi-sectoral land-use modelling outlined in Koomen et al. (2015)**,** local utility values are derived from combining information on 1) spatial drivers affecting the local suitability for supporting alternative land uses and functions; and 2) economic factors affecting the potential revenues and costs of different land-use activities. Local drivers include both exogenous (e.g., biophysical factors such as topography, climate and soil characteristics, availability of infrastructure networks, and zoning regulations) and endogenous drivers (accessibility, previous land-use and population patterns, and resulting neighbourhood land-use interaction effects). Together, endogenous and exogenous drivers determine the local suitability for different land uses and functions,

#### Specifying the Regional Demand Module

For this particular validation exercise, we have only focused on validating the allocation module, in order to distinguish allocation errors from quantity errors (sensu Pontius et al., 2018). The regional demand module was therefore specified by computing the total area of each simulated land use type and total population counts in each NUTS 3 region as observed in the reference maps during the period of 1990 and 2015. In particular, regional demand for urban land use and total regional population were computed for each region and time-step using the Global Human Settlement Layer (GHSL) data package (Florczyk et al., 2019) to represent observed urban land-use and population patterns in the years 1990, 2000 and 2015. The values for the 5-year time-steps in between were linearly interpolated. The regional demands for the other simulated land-use types were similarly determined, using the CORINE Land Cover (CLC) data series (Büttner et al., 2002, 2014) as a reference for 1990, 2000, 2006 (reference for simulated 2005 time-step) and 2012 (reference for 2015 time-step), excluding the gridcells classified as urban in GHSL, in order to not account for area that is classified as urban residential in the reference map.

#### Specifying the Local Allocation Module

Following the modelling framework proposed in Jacobs-Crisioni et al. (2017), the joint spatial allocation of population and land use in the LUISA model is based on an iterative procedure (Fig. 1), requiring the specification of local attractiveness for residential functions (Eq. B1 in the Supplementary Information), local population pressure (i.e. the degree to which changes in regional population lead to changes in local demand for residential land use; Eq. B3 in the Supplementary Information), local land-use suitability (i.e. the suitability of a location to support a particular land use, given its geographical characteristics and features; Eq. B4 in the Supplementary Information), and local land-use utility (Eq. B5 in the Supplementary Information). For the simulation of land use, we implement the discrete version of the utility-maximising spatial allocation algorithm proposed in Hilferink and Rietveld (1999). Once all land-use types (including urban residential land use) have been allocated so that all regional land-use demands are fulfilled, nighttime population (i.e. number of permanent inhabitants registered in a location) is then allocated according to the spatial algorithm introduced in Jacobs-Crisioni et al. (2017). The specification of this iterative procedure, and respective modelling equations and spatial allocation algorithms, are described in detail in Appendix B in the Supplementary Information. In the following we briefly describe the main steps in the specification of the allocation module for the validation exercise.

Local attractiveness for residential functions and local land-use suitabilities were empirically estimated separately for each simulated country based on observed population and land-use (change) patterns, respectively, using a set of spatial drivers as explanatory variables (see Appendix C in the Supplementary Information, for the complete list of variables). Local attractiveness for residential functions was estimated through ordinary least squares regression analysis, using the Global Human Settlement Layer (GHSL) population data series (Florczyk et al., 2019) as a reference for observed population patterns in 1990–2015. Residential attractiveness is translated into a measure of local population pressure, by taking into account the regional net population changes occurred between time-steps (positive or negative). Population pressure is then used as an explanatory factor for the land-use suitability of urban residential land use. Land-use suitability functions were estimated for all simulated land-use types through binomial logistic regression analysis. For the estimation of land-use suitability of residential land use, we used the time series of built-up densities registered in the GHSL data series (Florczyk et al., 2019) as a reference for urban land changes occurring between 1990 and 2015. The other simulated land-use types (i.e. industry and commerce, agriculture, forestry) were fitted using CLC 2012 (Büttner et al., 2014) as a reference to represent the presence of land cover.

Finally, land-use utilities were computed using the Net Present Value (NPV), a standard method for appraising long-term investments, such as those occurring when land is converted for a different use (Diogo et al., 2015; Koomen et al., 2015). NPVs are computed in a spatially explicit-way for each simulated land-use type, by taking into account their local land-use suitability and economic drivers affecting their economic viability, particularly their specific initial land-use conversion costs and the expected annual revenues and costs resulting from the use of land over time (see Section B.2 in the Supplementary Information). The revenues, costs, discount rates and time-horizons for the different land-uses were specified through a combination of literature review, available databases, official statistics and modelling results from specialised models (see Appendix D in the Supplementary Information, for a specification of these variables for the Netherlands, as an illustrative example). The calculated NPVs are not presumed to be exact estimations of land-use utility, but rather to provide a measure of the relative competitiveness among different land-use types in each location.

### Model Validation

A pixel-based map comparison was performed to assess the extent to which the allocation module is able to replicate the observed patterns of population and urban residential land-use change. The patterns of the other simulated land-use types (i.e. agriculture, forestry, industrial) were hereby not validated, in order to focus the validation exercise on evaluating the ability to jointly simulate urban residential land use and population in an integrated way. For each country, we computed a number of indicators assessing the performance of the model (see Sects. "Validating Urban Residential Land-Use Patterns" to "Multiple Resolution Validation").

Similarly to Pontius et al. (2004, 2008), we compared the performance of the LUISA model to two reference models in order to infer on its additional predictive power:

- *Null model*, i.e. a model predicting strictly static land use and population, i.e. no change in land-use and population patterns compared to the year 1990;
- *Random model*, which in this case was defined as an edge growth model that allocates urban residential land-use change as a non-uniform diffusion process beyond the settlement fringe of existing urban areas. For this purpose, in every time-step buffers were specified around the existing urban residential areas in a way that the total area of the buffers was equal to the total area of the observed land-use changes, with the buffer's radius being dependent on the settlement's morphology parameters. This model is thus comparable to the neutral model of landscape change proposed by Hagen-Zanker and Lajoie (2008) as a benchmark for the assessment of model performance. After the *Random* model allocates residential land use in concentric patterns, population is then allocated in the same way as the LUISA model over the generated land-use patterns.

Because population is allocated over the patterns of residential land use simulated in a previous step, the ability to correctly simulate population is, therefore, highly dependent on the ability to correctly allocate residential land use. Therefore, we produced an additional reference model – *LUISA model, simulation of population only — *in which the allocation of population was simulated over the patterns of urban residential land use as observed in the reference maps for the period of 1990–2015. This procedure allows to distinguish errors in the allocation of population that are inherent to the population allocation algorithm from errors that result from the misallocation of urban residential land use. All reference models described above were simulated in the LUISA modelling platform.

When land-use models are validated, their performance is typically evaluated over the whole extent of the simulated area, without distinguishing areas with different characteristics for which the model may perform differently (Pontius et al., 2008, 2018; van Vliet et al., 2016), for example, different types of urban morphological zones (e.g. cities, suburbs and rural areas). Therefore, we also evaluated the model performance separately according to different types of urbanised areas, as defined by the degree of urbanisation (DoU) typology (Dijkstra & Poelman, 2014). For this purpose, we used the DoU 2015 dataset from the GHSL data series (Florczyk et al., 2019), which classifies local administrative units (LAU), and respective gridcells, based on a combination of characteristics regarding geographical contiguity of urbanised areas and population density. Accordingly, three types of urbanised areas are distinguished (see Dijkstra & Poelman, 2014 for a more extensive description of the method to derive the degree of urbanisation typology):

- Cities: at least 50% of the LAU population lives in high-density clusters (i.e. contiguous gridcells with a density of at least 1,500 inhabitants per km.^2^ and a minimum population of 50,000);
- Rural areas: more than 50% of the LAU population lives in rural gridcells (i.e. gridcells outside urban clusters, which in turn are defined as clusters of contiguous gridcells with a density of at least 300 inhabitants per km^2^ and a minimum population of 5,000);
- Towns and suburbs: less than 50% of the LAU population lives in rural grid cells, and less than 50% lives in high-density clusters.

#### Validating Urban Residential Land-Use Patterns

To validate the simulated patterns of urban residential land-use change, three land-use maps were overlaid and compared for each country:

- a reference map of the initial time-step, i.e. GHSL 1990;
- a reference map of the final time-step, i.e. GHSL 2015;
- a simulated land-use map of the final time-step, i.e. urban residential land-use patterns in 2015 as simulated by LUISA, having GHSL 1990 as a starting point for the simulation.

We adapted the terminology of Pontius et al. (2018) to characterise the pixels in the model validation exercise (see also Table 1 for a summary):

- Misses (*M*): erroneous pixels due to observed change predicted as persistence. This can be due to not predicting the development of new urban residential areas (*M*_*1*_) or the occurrence of urban abandonment (*M*_*2*_);
- Hits (*H*): correct pixels due to observed change predicted as change. This can be due to correctly predicting new urban residential areas (*H*_*1*_) or correctly predicting the occurrence of urban abandonment (*H*_*2*_);
- False alarms (*F*): erroneous pixels due to observed persistence predicted as change; this can be due wrongly predicting the development of new urban residential areas (*F*_*1*_*),* or wrongly predicting the occurrence of urban abandonment (*F*_*2*_);
- Correct persistence (*P*): correct pixels due to observed persistence predicted as persistence; for the purpose of assessing the model‘s ability to simulate residential land use, we only take into account correct persistence of urban residential;
- Correct rejection (*R*): correct pixels due to observed non-residential land use predicted as non-residential.

**Table 1:** Pixel categories in the validation of simulated patterns of residential land use

	Observed 2015: Urban residential Simulated 2015: Urban residential	Observed 2015: Urban residential Simulated 2015: Other land use	Observed 2015: Other land use Simulated 2015: Urban residential	Observed 2015: Other land use Simulated 2015: Other land use
Observed 1990: Other land use	H_1_	M_1_	F_1_	R
Observed 1990: Urban residential	P	F_2_	M_2_	H_2_

Based on these categories, we computed a series of indicators of land-use (change) agreement for each country. Firstly, we evaluated the ability of the model to correctly simulate the establishment of new urban residential areas and the occurrence of urban abandonment. For that purpose, we computed the producer’s accuracy (*PA*, i.e. the share of pixels with observed land-use change that was correctly simulated), and the user’s accuracy (*UA*, i.e. the share of pixels with simulated land-use change that was correctly simulated) as adapted from Pontius et al. (2008):

1 $$PA= \frac{H}{H+M}$$

2 $$UA= \frac{H}{H+F}$$

Furthermore, we also assessed the ability of the model to simulate the persistence of residential land use, by computing the share pixels with well-predicted persistence (*%P*) in relation to the pixels with observed persistence (adapted from Diogo et al., 2014):

3 $$\%P= \frac{P}{P+{F}_{2}}$$

Finally, we assessed the overall model performance (*MP*) in simulating residential land use, i.e. the extent to which the simulated residential land-use patterns correspond to those observed in the reference map (adapted from Diogo et al., 2014):

4 $$MP= \frac{{H}_{1}+P}{{H}_{1}+P+{M}_{1}+{F}_{2}}$$

Hence, *PA* and *UA* assess the ability of the model to simulate urban residential land-use change; *%P* assesses the ability of the model to replicate observed inertia of urban residential land use; and *MP* assesses the ability of the model to reproduce the overall pattern of observed urban residential land use.

#### Validating Population Patterns

To validate the simulated population patterns, two population maps were overlaid and compared for each country:

- a reference population map of the final time-step, i.e. GHSL 2015 population;
- a simulated population map of the final time-step, i.e. population patterns in 2015 as simulated by LUISA, having GHSL 1990 population map as a starting point for the simulation.

It should be noted that GHSL population maps are based on dasymmetric mapping methods of regional population statistics, and not real observations. Nevertheless, we consider it to be the best available dataset for representing population distributions patterns across the whole EU, and thus we find it appropriate for validating simulated population patterns. Based on this comparison, we computed for each country the degree of correspondence (*DoC*), an indicator of agreement between simulated and observed patterns for continuous variables (adapted from Loonen & Koomen, 2009):

5 $$DoC=100\%*\left(1- \frac{\sum_{i=1}^{n}\frac{\left|{Spop}_{i}-{Opop}_{i}\right|}{2}}{\sum_{i=1}^{n}{Opop}_{i}}\right)$$

where:

*Spop*_*i*_: is the amount of people allocated by the model in gridcell *i* in 2015;
*Opop*_*i*_: is the observed amount of people in gridcell *i* in 2015;
*N*: is the total amount of land gridcells within a country.

The *DoC* is closely related to the mean absolute percentage error, a commonly used indicator to measure the accuracy of forecasting methods. However, the absolute difference between simulated and observed amount of people is hereby divided by 2, to avoid double-counting of misallocated population. The *DoC* ranges from 0 (i.e. none of the simulated amount of people is allocated in the corresponding grid-cells with observed population) to 100% (i.e. the simulated amount of people is equal to the observed amount in every grid-cell). In addition, we also computed the mean bias error (*MBE*) in order to identify the systematic error of the model to under- or over-forecast population:

6 $$MBE= \frac{\sum_{i=1}^{N}{Spop}_{i}-{Opop}_{i}}{N}$$

where:

*MBE*: is the the mean bias error;
*N*: is the number of gridcells in a country with population observed in 2015.

#### Multiple Resolution Validation

A pixel-by-pixel comparison can be somewhat misleading in terms of assessing the ability to correctly allocate land use and population. For instance, the model might be allocating a certain land use or amount of people in the surroundings but not exactly on the correct grid-cells, leading to the conclusion that land use and/or population is being wrongly allocated, while in fact the model is producing sensible patterns. A multiple resolution procedure was therefore applied to investigate how the scale of assessment influences the scores of the indicators of agreement. An expanding sampling window was used to gradually decrease the resolution of the comparison, in order to quantify the indicators as the resolution of measurement becomes coarser. Instead of comparing the maps on a pixel-by-pixel basis, we counted the number of pixels with simulated residential land use within the whole sampling window and compared it with the number of pixels with residential land use within the same window in the reference map. A comparable procedure was introduced to assess the indicators of agreement for population, but in this case, we counted the total amount of population allocated in the pixels within the sampling window, and then compared it with the amount of population observed in the reference map within the same window. Finally, a weighted average of the indicators at different window sizes is determined to summarise the overall fit. The weighted average is calculated so that the greater window size, the smaller is the weight given to the respective indicator on the average, as follows (adapted from Costanza, 1989):

7 $$WI=\frac{\sum_{w=1}^{n}{I}_{w}*{e}^{-k(w-1)}}{\sum_{w=1}^{n}{e}^{-k(w-1)}}$$

where:

*WI*: is a weighted average of the indicator of agreement (i.e. *WPA*, *WUA*, *WMP* or *WDoC*) over all window sizes;
*I*_*w*_: is an indicator of agreement (i.e. *PA*, *UA*, *MP* or *DoC*) when the sampling window is of linear dimension *w*;
*n*: is the sampling window with largest linear dimension considered in the multiple resolution validation (in this particular case, *n*=100, i.e. 10.000 ha);
*k*: is a constant (0.1 as default value) to determine the weight that is given to small sampling windows in comparison to larger ones.

## Results

### Urban Residential Land-Use Patterns

**Fig. 2:**
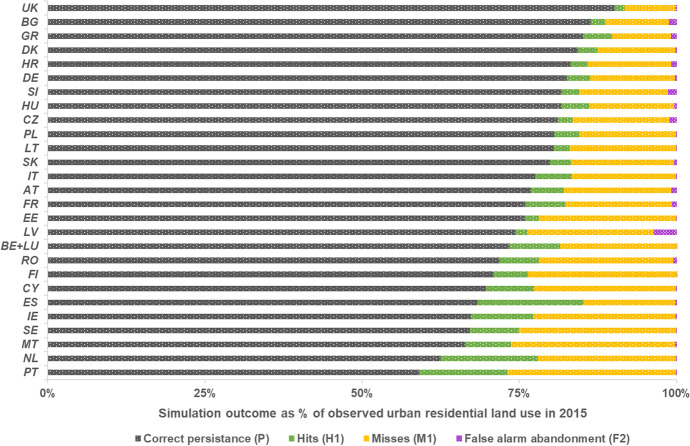
Share of correct persistence, hits and misses of urban residential land use and false alarms of urban abandonment as simulated by LUISA, in relation to the total residential land use area observed in 2015

The performance of the model in simulating residential land use is determined by the ability to correctly simulate both land-use persistence and change (*P* and *H1* in Fig. 2). Persistence of residential land use appears to be well captured in the model, with all countries achieving a share of correctly simulated persistence in relation to observed persistence (*%P*) higher than 98%. Over-optimization of land use seems to somewhat occur in several countries though, leading the model to overestimate the occurrence of urban abandonment (*F2* in Fig. 2), and re-allocating these residential areas somewhere else (thus also contributing to false alarms of residential land use, i.e. *F1*). However, this amounts to only 0.3% of the total urban residential area observed in EU27 + UK in 2015, with the highest shares being observed in Estonia (3.7%), Slovenia (1.4%), Bulgaria (1.2%) and Czechia (1.1%). Hence, producer’s accuracy (*PA*) and user’s accuracy (*UA*) values are roughly the same for all simulated countries.

**Fig. 3:**
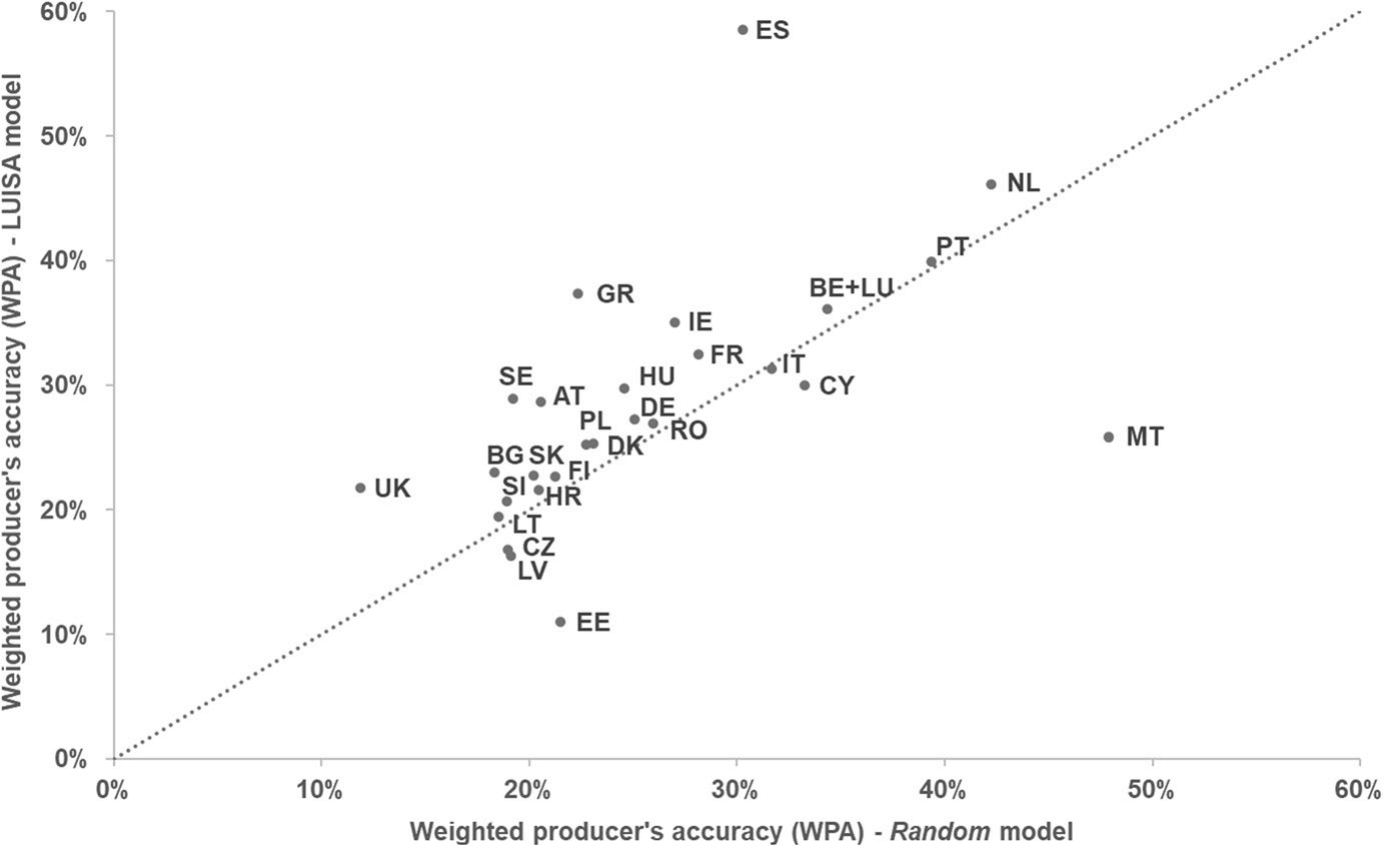
Weighted producer’s accuracy (WPA) in reproducing observed residential land-use change patterns in 1990–2015, for the LUISA and *Random* models

In terms of correctly allocating land-use change, the LUISA model does not perform equally well across countries (Fig. 3). In most cases, it achieves a weighted producer’s accuracy (*WPA*) between 20 and 40%. The model performs relatively well in the Netherlands (*WPA* = 46%) and Spain (*WPA* = 59%), but particularly poorly in Estonia (*WPA* = 11%). Figure 3 also shows that the LUISA model has, to some extent, a comparable performance to the *Random* model, i.e. the model simulating urban residential land-use change as a diffusion process on the settlement’s fringes. This seems to indicate that the LUISA’s land-use allocation module tends to favor the formation of concentric urban clusters. For most countries the LUISA model still provides additional predictive power compared to the more mechanistic *Random* model, particularly in Spain (+ 28 percentage points (pp)) and Greece (+ 16 pp), with the exceptions being Czechia (-2 pp), Latvia (-3 pp), Cyprus (-3 pp), Estonia (-10 pp) and Malta (-21 pp).

**Fig. 4:**
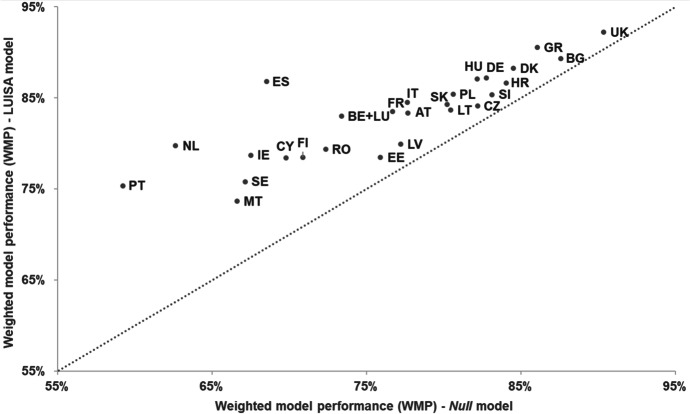
Weighted model performance (*WMP*) in reproducing observed residential land-use patterns in 2015, for the LUISA and *Null* models

Overall, the model is able to reproduce the observed land-use patterns reasonably well, with almost all countries showing a weighted model performance (*WMP*) larger than 75% (Fig. 4). It appears to perform particularly well in United Kingdom (*WMP* = 92%), and Greece (*WMP* = 91%), mostly due to the relatively high share of observed persistence that was well predicted. The LUISA model outperforms the *Null* model in all countries, i.e. it provides additional predictive power compared to not simulating land use at all. It performs particularly well in the Netherlands (+ 17 pp compared to the *Null* model), Portugal (+ 16 pp) and Spain (+ 18 pp), i.e. countries with a significant relative increase in urban residential land use between 1990 and 2015 and where the LUISA model showed a good ability to correctly allocate urban land-use change.

**Fig. 5:**
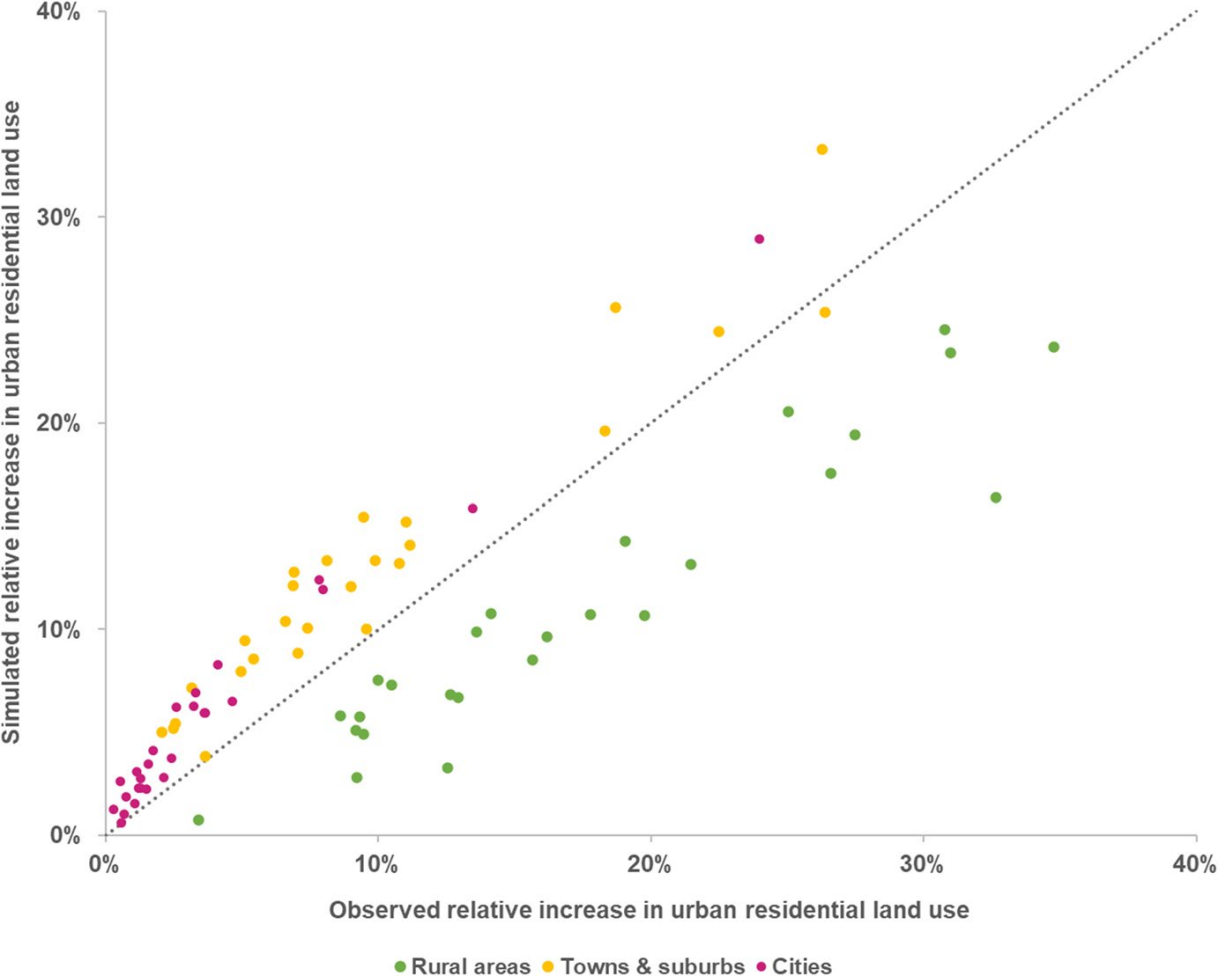
Relative net change of residential land-use area in the period of 1990–2015, both observed and simulated per country, according to the degree of urbanisation

Figure 5 shows the relative net change of residential land-use area during the period of 1990–2015 for each country, according to the degree of urbanisation of the locations where the establishment of new urban residential areas occurred (both observed and simulated). We can observe that the model is systematically biased towards underestimating the development of urban residential land use in rural areas (-6 pp, on average in EU27 + UK), and conversely overestimates the growth of urban residential land use in cities (+ 2 pp, on average) and in towns and suburbs (+ 3 pp, on average).

**Fig. 6:**
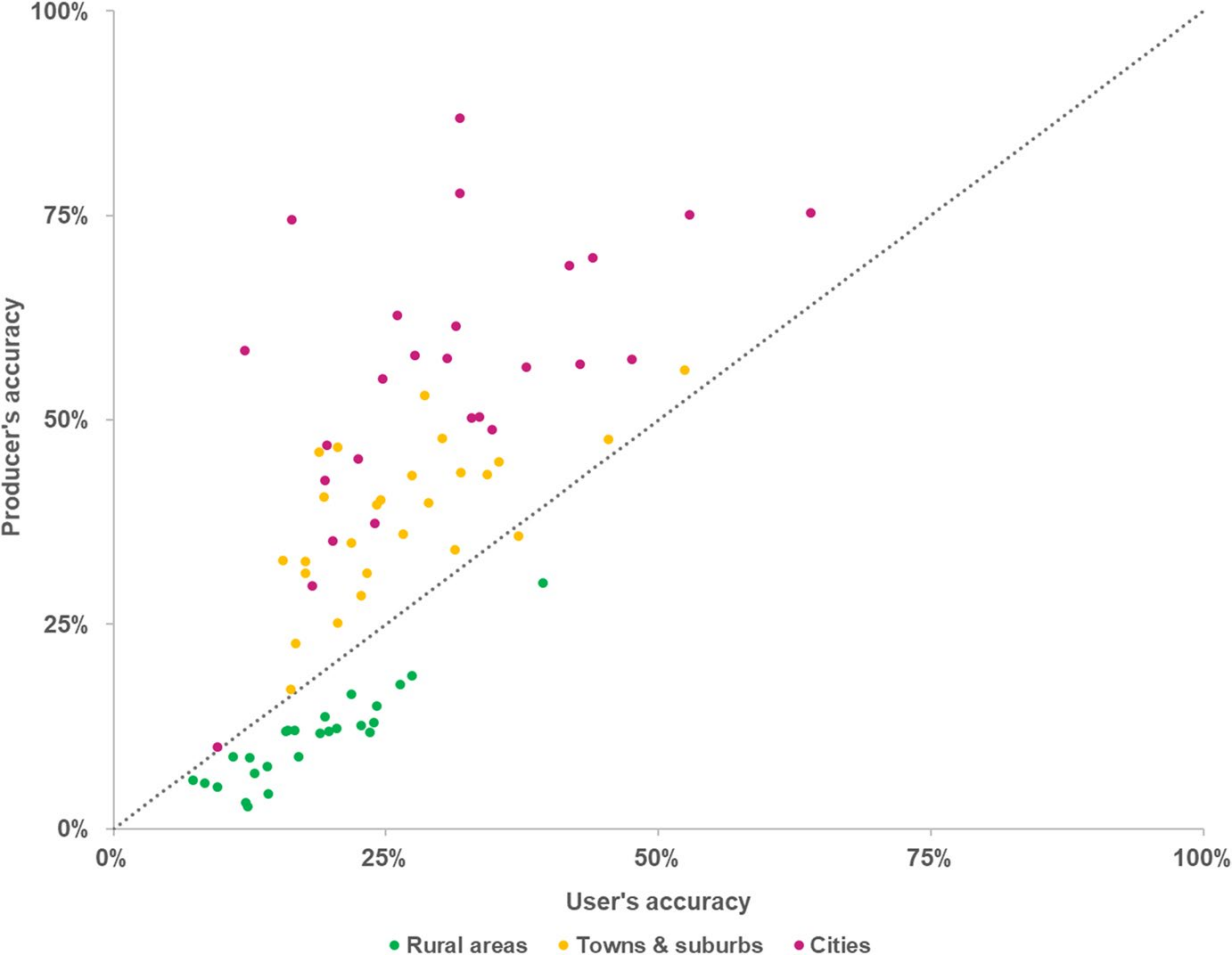
Producer’s accuracy (*PA*) and user’s accuracy (*UA*) per country, according to the degree of urbanisation

**Fig. 7:**
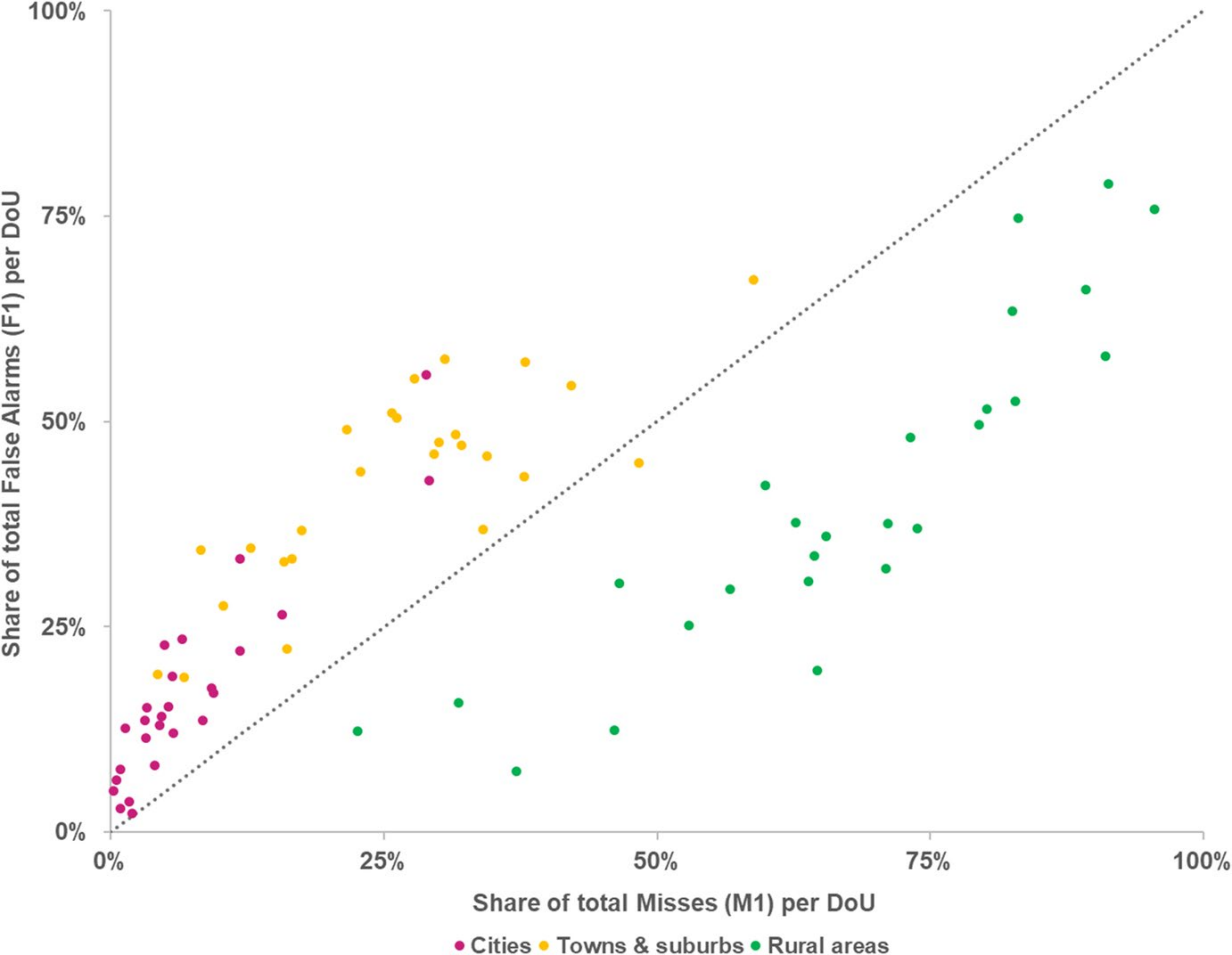
Share of total occurrence of false alarms (*F1*) and misses (*M1*) per degree of urbanisation, in relation to total false alarms and misses, in each country

The model’s accuracy (both *PA* and *UA*) in correctly allocating new urban residential areas is consistently higher in cities and in towns and suburbs areas than in rural areas (Fig. 6). Overall, *PA* in EU27 + UK is 56% in cities, 35% in towns and suburbs, and 11% rural areas. Furthermore, *PA* is systematically higher than *UA* in cities (on average, + 21 pp) and in towns and suburbs (+ 9 pp). In other words, the share of observed land use that is correctly predicted is higher than the share of simulated land use that is correctly predicted, with the overestimation of urban residential land use in these areas resulting in a relatively higher occurrence of false alarms in relation to misses (Fig. 7). The opposite relationship is observed in rural areas, i.e. *UA* is higher than *PA* (on average, + 9 pp), due to the substantially larger share of misses in relation to false alarms. *PA* in cities is high even in countries that have shown an overall low *WPA*, e.g. Estonia (75%) and Lithuania (78%). This implies that the model is able to correctly identify the locations where urban growth takes place in cities, even in the countries where the model has an overall poor performance. However, and particularly for those countries, that also implies a relatively lower *UA* in cities – i.e. the model might correctly predict the observed establishment of residential land use in cities, but that comes at the cost of also wrongly predicting a relatively large share.

**Fig. 8:**
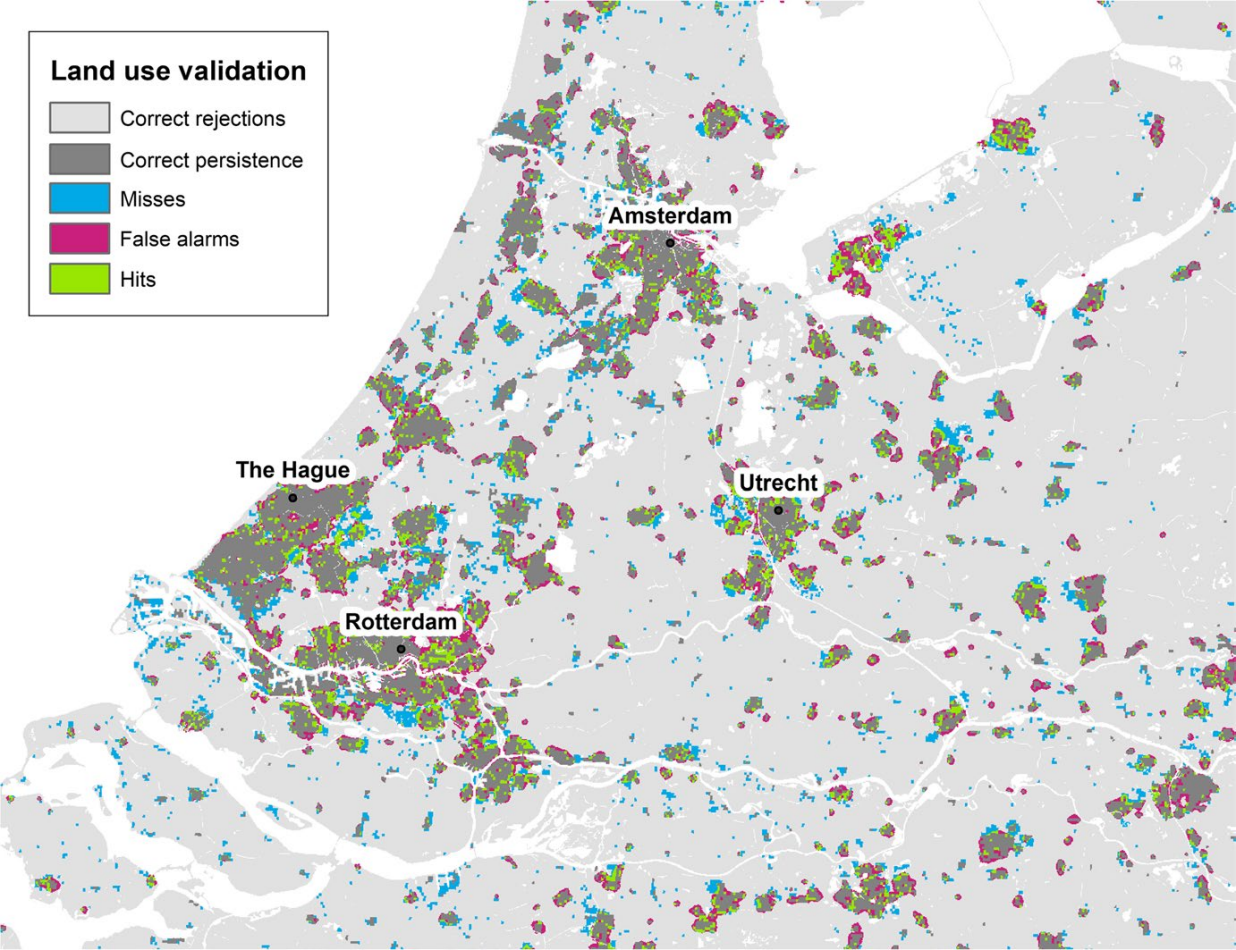
Pixel-by-pixel comparison of simulated and observed patterns in the Randstad region, the Netherlands

As a means to illustrate the model’s behaviour and ability to simulate urban residential land-use development, Fig. 8 shows a pixel-by-pixel comparison of the observed and simulated patterns in the so-called Randstad region in central-western Netherlands. This region consists of a large group of adjacent metropolitan areas including the four largest Dutch cities (Amsterdam, Rotterdam, The Hague and Utrecht) and their surrounding (peri-)urban and rural areas. We can observe that combinations of hits, false alarms and misses seem to occur together within existing urban conglomerations and on their fringes. This implies that, despite the errors at a fine, pixel-by-pixel resolution, the model is able to capture relatively well the aggregated spatial pattern resulting from the growth of existing cities, towns and suburban areas. However, we also see a relatively large number of scattered misses occurring in rural areas, particularly on the fringe of existing small conglomerations and in newly formed ones.

**Fig. 9:**
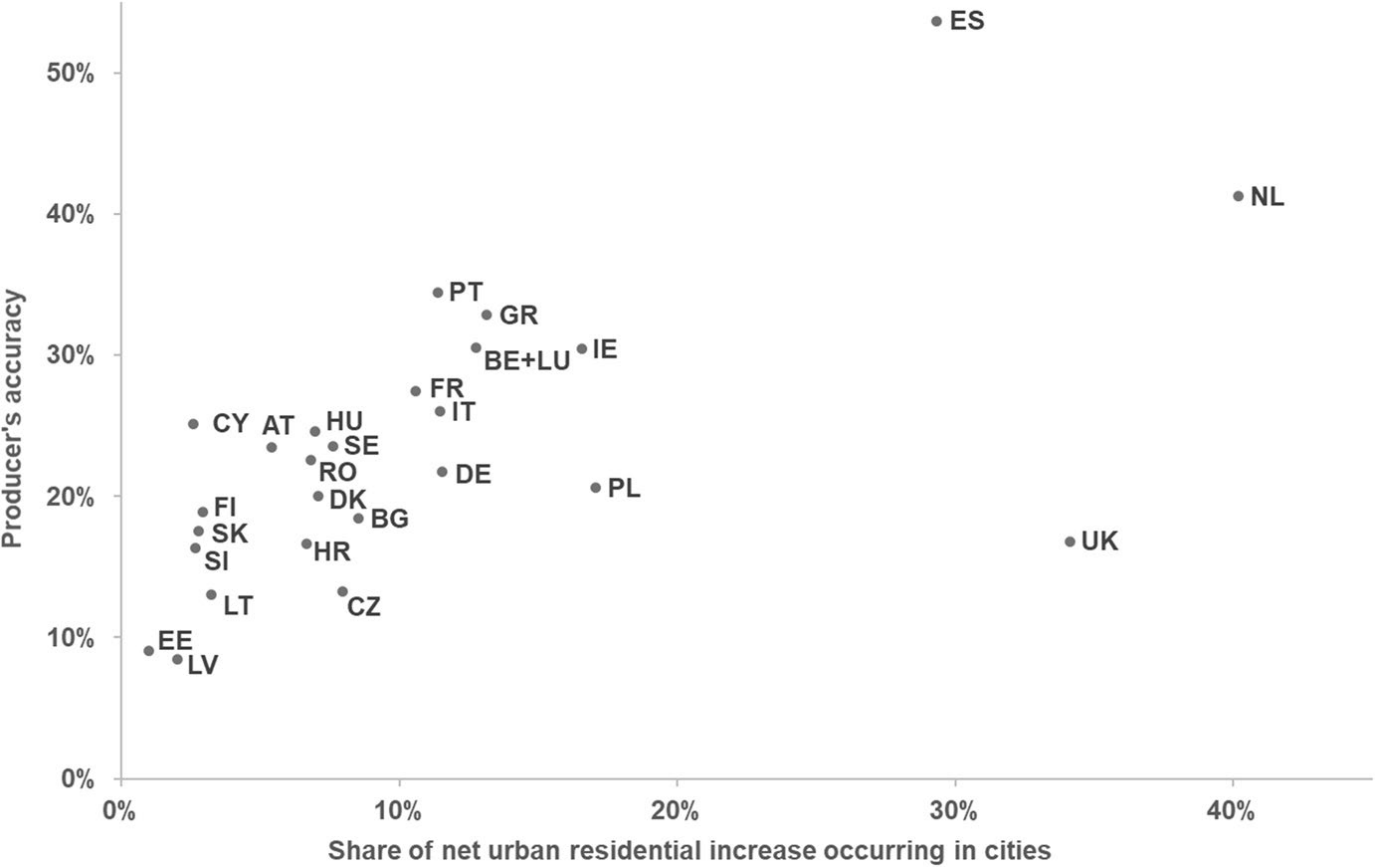
Producer’s accuracy as a function of the share of net urban residential land-use change occurring in cities

These observations seem to underpin the previous observations on the relatively poorer ability of the model to reproduce land-use patterns in rural areas. In fact, we can observe that differences in model performance across countries seem to be mainly explained by the predominant pattern of urban development taking place in each country, with higher *PA* values being largely linked to high shares of net residential land-use change occurring in cities (Fig. 9) and lower shares in rural areas. We can therefore conclude that the LUISA model seems to favor the formation of compact, large urban clusters in cities and existing towns and suburbs, while somewhat failing to capture the emergence of new isolated suburban neighbourhoods and small towns in rural areas.

### Population Patterns

**Fig. 10:**
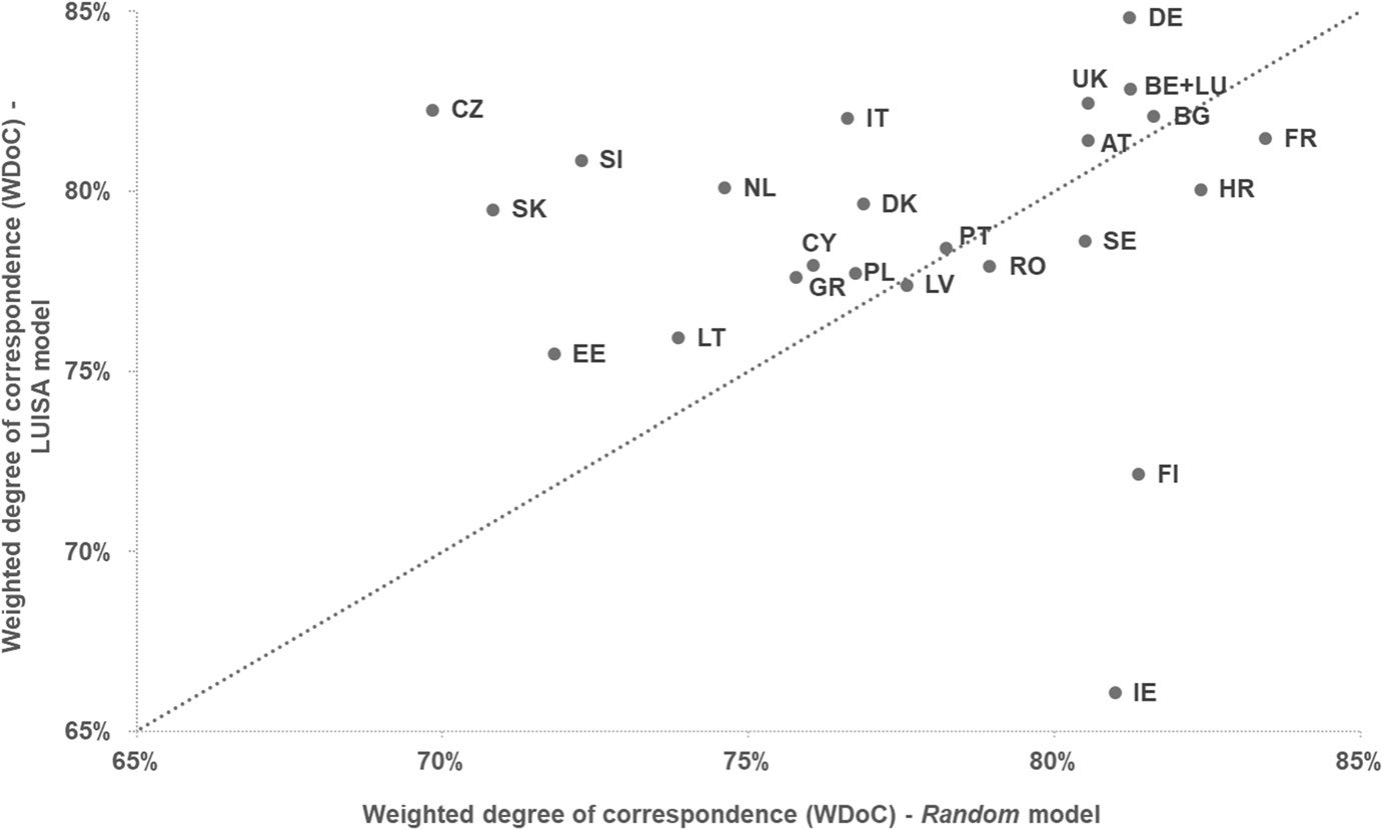
Weighted degree of correspondence (*WDoC*) in reproducing observed population patterns in 2015, for the LUISA model and the *Random* model

**Fig. 11:**
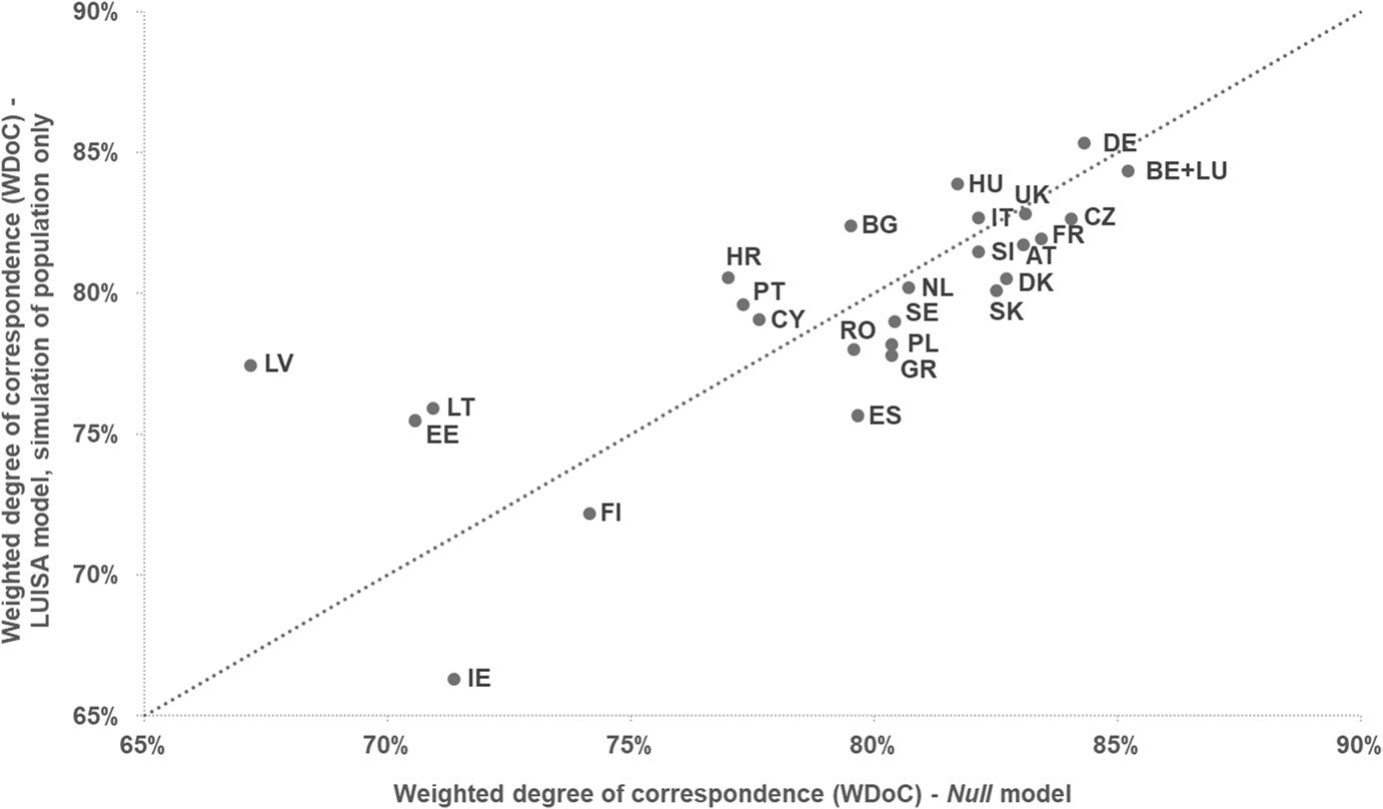
Weighted degree of correspondence (*WDoC*) in reproducing observed population patterns in 2015, for the *LUISA model, simulation of population only* and the *Null* model

Figure 10 shows that, regarding the ability to correctly simulate population patterns, the LUISA model achieves a weighted degree of correspondence (*WDoC*) between 75 and 85% for most countries, except for Finland (72%) and Ireland (66%). The LUISA model outperforms the *Random* model in several countries, but not all. Specifically, for Latvia, Romania, Sweden, Croatia, France, Finland and Ireland allocating population over the concentric urban land-use patterns produced by the *Random* model generates population patterns that are more in agreement with the observed ones, than those generated by the LUISA model. By comparing Figs. 10 and 11, we can see that the *LUISA model, simulation of population only* (i.e. the model with which population is simulated over the observed land-use patterns) performed only slightly better than the LUISA model (+ 0.8 pp on average). This seems to indicate that errors in the allocation of population can be somewhat explained by misallocation of urban residential land use, but not entirely. In addition, Fig. 11 also shows that the *Null* model performs best for a large number of countries, i.e. simulating population changes introduces more errors than assuming no population change. No clear relationship could be found between differences on the ability of the model to correctly allocate population and the characteristics of the respective countries and regions, for example, in terms of NUTS 3 region size, predominance of urban areas, magnitude of population change (either absolute or relative), direction of population change (i.e. net decrease or increase), or share of population (change) per degree of urbanisation. We conclude that even though the LUISA model performs overall reasonably well in reproducing observed population patterns, the current country-specific empirical functions of population pressure and distribution do not seem to fully capture all the different drivers and processes of population distribution change operating across the various countries and regions in the EU27 + UK.

**Fig. 12:**
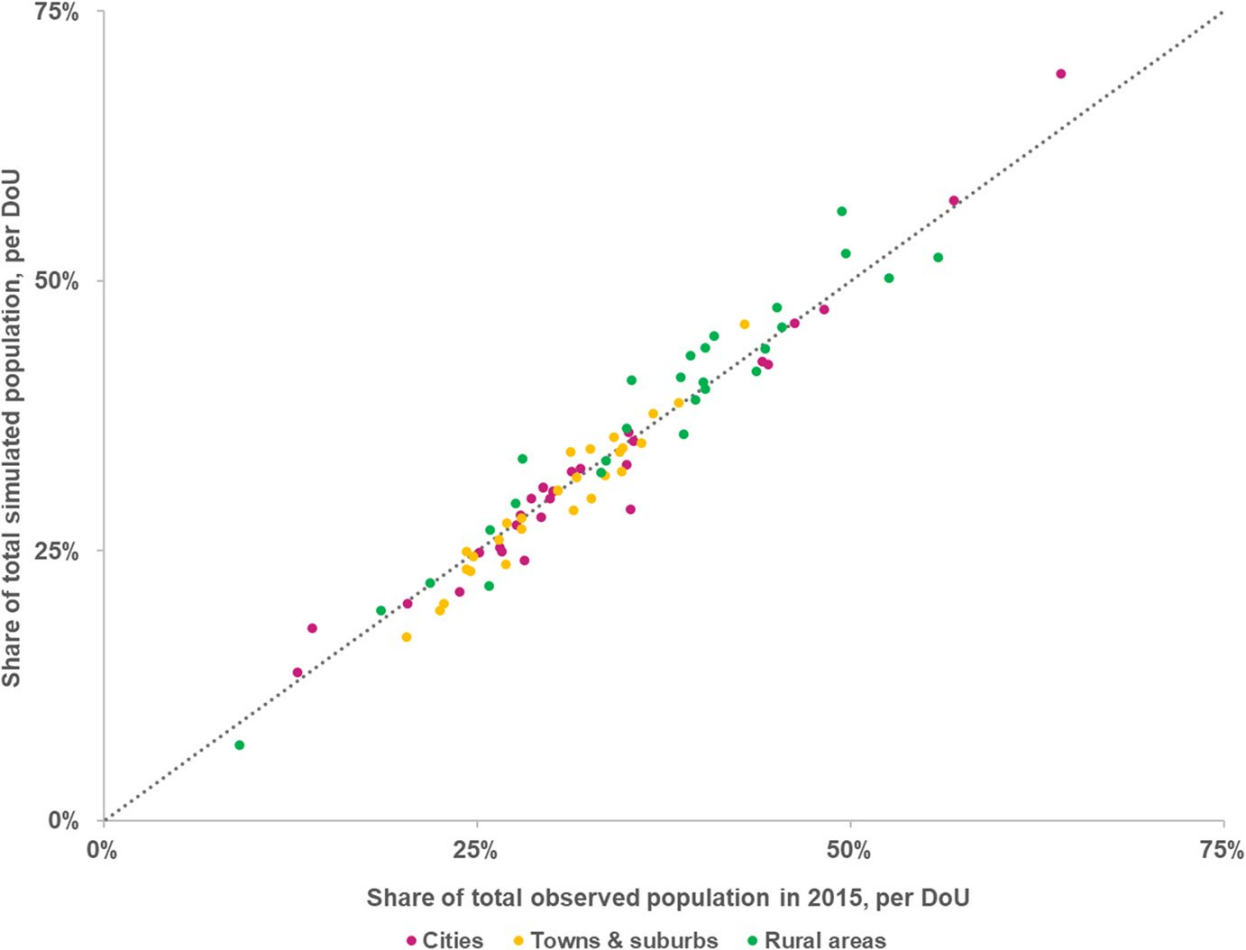
Share of total country population as simulated by the LUISA model in relation to the share of total country population observed in 2015, per degree of urbanisation

In addition, we found that overall the LUISA model tends to slightly overestimate the number of people living in rural areas (+ 0.8 pp overall when comparing simulated with observed rural population in EU27 + UK, see Fig. 12) while underestimating population in cities and in towns and suburbs (-0.2 and -0.6 pp, respectively). However, in a few countries (e.g. Belgium, Denmark, Slovenia and Slovakia), the opposite relationship could also be observed.

**Fig. 13:**
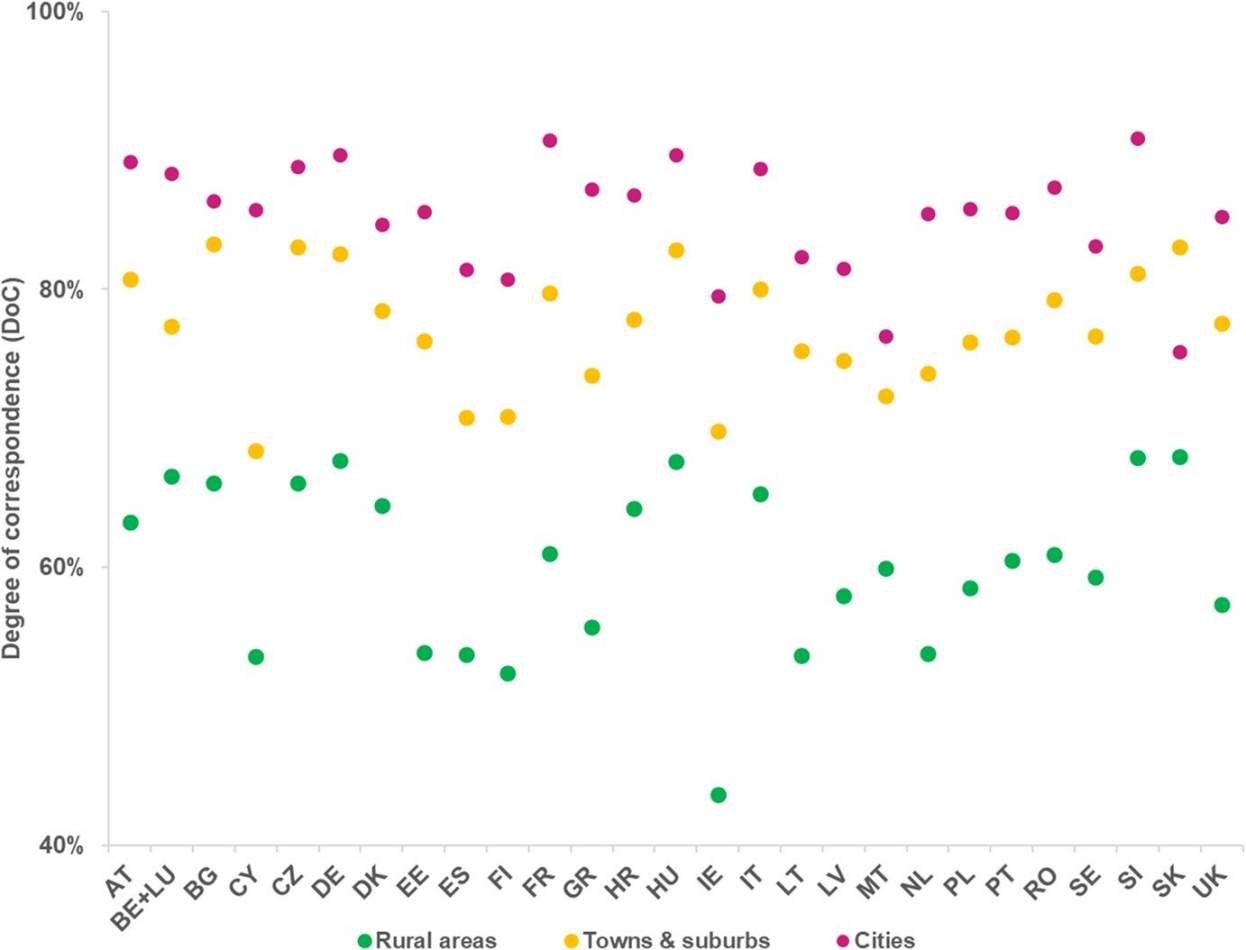
Degree of correspondence (*DoC)* of simulated population, according to the degree of urbanisation

**Fig. 14:**
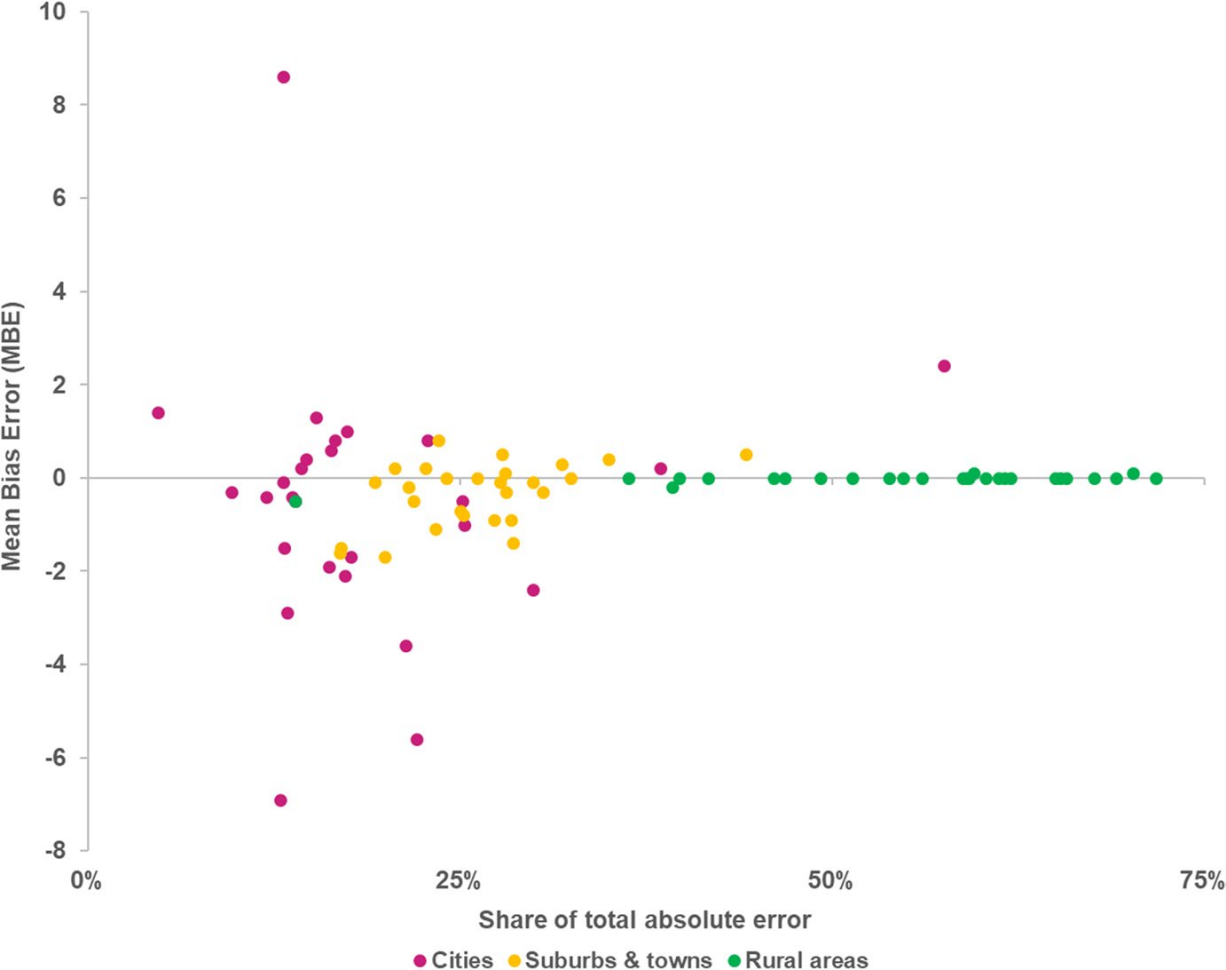
Mean bias error (*MBE*) per degree of urbanisation, and share of absolute total error per degree of urbanisation, of simulated population in each country

Figure 13 shows that the LUISA model performs differently in allocating population depending on the degree of urbanisation of observed land use. It consistently achieves a relatively higher *DoC* in cities (roughly between 80 and 90%) and in towns and suburbs (roughly between 70% and 85), compared to rural areas (lower than 70%). One can also observe in Fig. 14 that, although the mean bias errors (*MBE*) in rural areas are of very small absolute magnitude (on average, -0.01 people), they consistently account for the majority of the total absolute errors in a simulated country. This result implies that even though individual allocation errors in cities and in towns and suburbs are on average much larger, errors in rural areas are much more frequent and widespread.

**Fig. 15:**
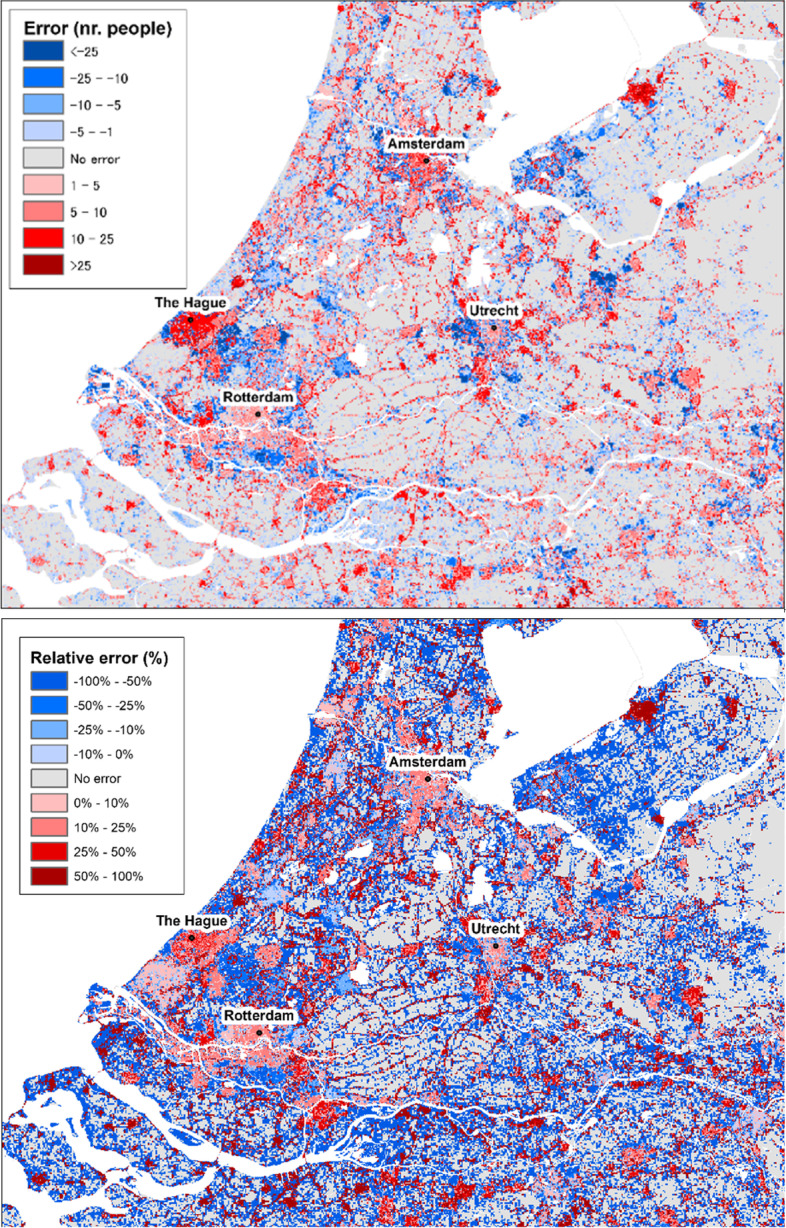
Absolute (above) and relative error (below) of the simulated population distribution in the Randstad, the Netherlands

Figure 15 shows a pixel-by-pixel comparison of observed and simulated population patterns in the Randstad region in the Netherlands, as a means to illustrate the model’s behaviour and ability to correctly allocate population. One can confirm that, although the allocation errors in rural areas are of small absolute magnitude (particularly when compared to those in large urban conglomerations), the relative errors are significantly large and distributed over a considerable area extent, both in towns (where they are largely overestimated) and sparsely distributed communities (where they are largely underestimated).

This seems to indicate that the model has a tendency to redistributing people from low density rural areas to residential clusters with good accessibility, resulting on overestimation of population in the core of urban conglomerations and well-connected towns, and conversely underestimation of the number of people living on more sparsely distributed rural communities. These findings also underpin the relatively lower model performance in rural areas (see Fig. 13). In addition, they also provide an explanation for the *Null* model outperforming the LUISA model in many countries (Fig, 11): the aggregated errors introduced by the model over-redistributing people from rural areas are so large that they surpass those in the *Null* model resulting from not accounting for the net regional population changes. On the other hand, this redistribution mechanism seems to capture relatively well the outcomes of negative net population change processes, e.g. Bulgaria, Croatia, Hungary, Estonia, Latvia, Lithuania, with the model outperforming the *Null* model in these cases.

**Fig. 16:**
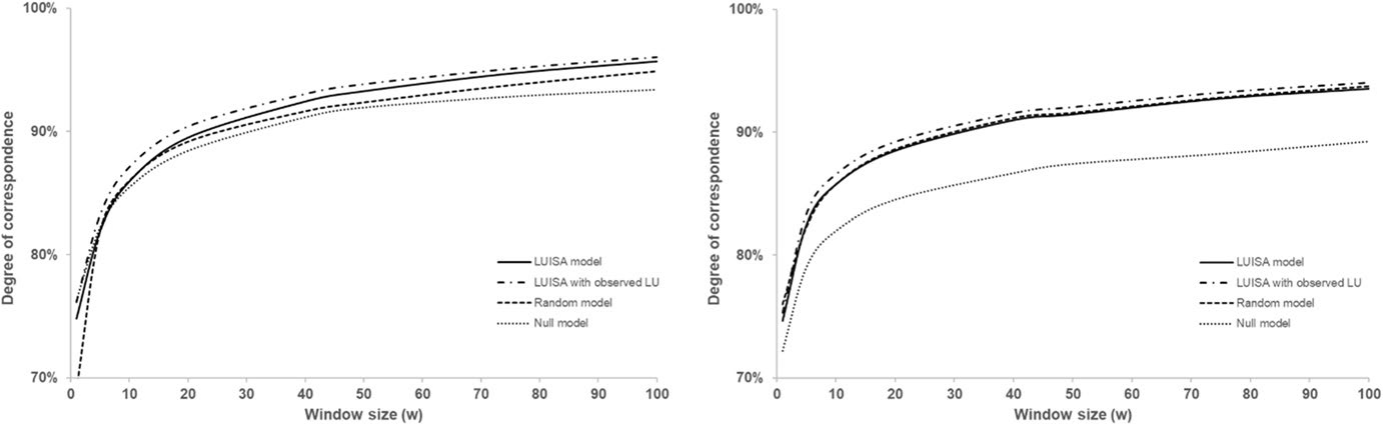
Degree of correspondence (*DoC*) at multiple resolutions for the Netherlands (left) and Croatia (right)

Despite the errors introduced at fine resolution by the dynamic population redistribution, the *DoC* consistently increases for larger window sizes, with the LUISA model outperforming the *Null* and *Random* models at the aggregated level for window sizes larger than 10 (i.e. > 1 km2), both when net population increases (e.g. as in the Netherlands) and decreases (e.g. as in Croatia), as illustrated in Fig. 16. Hence, we can conclude that the LUISA model is able to overall provide additional predictive power for projecting population distributions in aggregated administrative units (e.g. at the municipal level), compared to both *Null* and *Random* models.

## Discussion

The validation provided critical insights on the capabilities and limitations of the LUISA model to inform on future population density and land-use developments in the EU27 + UK. In terms of land-use modelling accuracy, the LUISA model showed a performance range comparable to that observed on previous validation exercises comparing different land-use modelling applications (see e.g. Pontius et al., 2008, 2018). Validating the model in different countries allowed evaluating its ability to reproduce observed land-use change patterns and processes across a diverse set of biophysical and socio-economic contexts. Interestingly, the validation results showed that, even though the same methods and data sources were uniformly applied in every simulated country, the ability of the model to correctly reproduce land use and populations patterns can still vary widely depending on the context. Pontius et al. (2008) had previously reported that the model’s ability to predict land-use change can be substantially hindered when predicting small amounts of net change, i.e. the models are more likely to predict land-use change accurately when the amount of observed net change is large. Our validation showed, however, that the amount of net change is not necessarily a determinant of model performance, but rather the location and overall pattern of change observed in the simulated region. In particular, the model performed consistently well in urban areas characterized by compact urban growth in cities and suburbs, but poorly where residential development occurred predominantly in scattered patterns across rural areas, irrespective of the amount of net change.

The model showed a tendency to simulate residential and population patterns towards the emergence of compact, densely populated and highly accessible urban conglomerations.Often such patterns did not entirely correspond to the ones observed in reality. A number of reasons may explain such model behaviour. With regard to the biased allocation of residential land use, a likely explanation comes from the fact that, in the taken modelling approach, different segments of the residential market were lumped together into one generic residential land-use type, thus failing to accurately capture the emergence of isolated peri-urban residential development observed in Europe (EEA-FOEN, 2016). Ideally, different segments of the residential land-use market should be modelled according to their own specification. This would require a substantial modification of the applied allocation algorithm in order to allow multiple land-use types satisfying one generic land-use demand. This is currently done, for example, in the CLUMondo (Van Vliet & Verburg, 2018) and Ruimtescanner XL (ObjectVision, 2020) models.

The same segmentation of preferences would have to be taken into account in the population allocation as well, given that the model currently considers population to be uniform. In addition, the model implicitly conflates historic population density with local attractiveness for residential functions. To a large extent, this is an oversimplification on the range of households’ residential preferences and choices, in respect to housing amenities and costs. This assumption can be particularly problematic when considering that informal settlements and social housing neighbourhoods are often densely populated but largely lacking amenities (Beer et al., 2003; Visagie & Turok, 2020). In reality, population age structure and differences between age groups in terms of preferences (e.g. open-space, availability of services, noise), household size and income may contribute to the emergence of different housing market segments. In addition, different age groups may also hold distinct mobility patterns. Recent developments in breaking down local population distributions by age classes (Jacobs-Crisioni et al., 2020) and identifying intraday and monthly population variations (Batista e Silva et al., 2020) may provide promising avenues for addressing these issues and developing modelling approaches that explicitly account for these processes.

In addition, while urban residential and other land-use types are represented in the model as homogenous, mutually exclusive classes, in reality a large share of urban land-use changes in Europe occurs in very small incremental changes rather than large-scale conversions, particularly in rural areas (Van Vliet et al., 2019). Although residential land use was simulated at a relatively fine resolution, it is likely that a large share of rural residential areas remained unrepresented in the model, during both the specification of the local suitability functions and simulation of land use. This may partially explain the poorer performance of the model in rural areas, and at the same time, the improved ability to predict the establishment of urban residential land use in cities, given that this type of dichotomous classification is better suited to capture high-density urban areas. These findings suggest that the calibration and simulation of residential land use can be improved by replacing such dichotomous classifications by more nuanced representations of land use, including e.g. mosaic classes or continuous variables indicating the share of land use in a gridcell.

Not accounting for variations in e.g. local-specific land prices, intensity-specific investment costs and housing prices may also partly explain the model behaviour. Land prices in cities are, in general, substantially higher than in the surrounding rural areas. Likewise, the costs of constructing high-rise buildings also differ substantially from those incurred in the construction of low-intensity residential areas. Real estate developers can be expected to take into consideration trade-offs between local attractiveness factors that contribute to the valuation of houses (and consequently their expected gross revenues) and development costs. Depending on local-specific land prices and construction costs (and also on the demand for different housing market segments, see previous issue), it might be more attractive, for example, to invest in the development of low intensity residential areas in locations with less amenities (thus, providing lower gross revenues per unit of land) but that also involve lower costs. These limitations may also contribute to the model over-optimizing land-use in relation to only a few particular land-use suitability factors (e.g. accessibility and neighbourhood population density), with the dynamic population allocation mechanism then reinforcing this model behaviour as a positive feedback, leading to the emergence of the simulated compact urban patterns. Variability on these factors could be incorporated by considering different magnitude ranges of costs and revenues, for example, according to the degree of urbanisation (which in turn, could be dynamically determined based on the simulated population and residential land-use patterns).

The spatial allocation algorithm implicitly assumes that allocating land to the uses maximising local utility delivers the greatest overall utility (Fujita, 1989; Martínez, 1992), while there is evidence that such free market mechanisms often lead to market failures in the land system (Cheshire, 2009, 2013). Spatial planning is both an important governance instrument for addressing these failures and a key driver of urban development patterns (Claassens et al., 2020; Hersperger et al., 2018), but it is currently not incorporated in the LUISA modelling framework. Given the apparent stochastic nature of the establishment of new towns, the inclusion of e.g. detailed maps representing municipal land-use planning regulations could contribute to improve the model performance, particularly in rural areas where it is most challenging to explain observed change patterns. However, harmonised digital datasets of spatial plans are currently lacking at the European level, in part also due to the inherent vagueness of strategic spatial planning (Hersperger et al., 2022). Therefore, while we fully endorse efforts for better embedding land-use modelling within the spatial planning domain, we argue that such efforts are beyond the scope of a large-scale modelling tool such as the LUISA platform. Instead, we contend that this should rather be pursued in the development of modelling applications for in-depth case studies in relatively small extent areas (e.g. at the metropolitan area level, see Domingo et al., 2021).

The LUISA model relies on a complex chain of modelling steps, requiring the integration of a large number of specialised datasets from different sources in order to calibrate the demand and allocation modules. Despite considerable efforts for coherently harmonising the entire knowledge base (see e.g. Baranzelli et al., 2014; Batista e Silva et al., 2016; Jacobs-Crisioni et al., 2017; Lavalle et al., 2013), errors may be inevitably introduced when processing the different datasets, not to mention the accuracy limitations inherent to each one of them. This makes it very challenging to assess the magnitude effect of error propagation in the model performance, and to distinguish these type of errors from those emanating from faulty assumptions or specifications in the modelling framework. Additional efforts should therefore be made to coherently evaluate the implications on the model behaviour and performance resulting from the data quality limitations of each individual dataset, and potential issues arising from the combined use of heterogeneous databases.

## Conclusions

Models represent simplified versions of reality, and as such, developing a model entails evaluating trade-offs and making decisions about the appropriate degree of abstraction and generalisation (Magliocca et al., 2015), i.e. which details from reality to include or exclude, and how to represent them, in order to provide a sensible representation of the system at hand. Such decisions are usually informed, for example, by the intended level of detail and extent of the modelled system, data availability for representing the considered system properties, and the purpose for which the model is developed. The model validation presented in this article appeared to be particularly helpful in revealing which aspects of land-use and population change appeared to be well captured by the modelled approximations, and which ones not. Not only does this provide policy-makers with a better understanding on how to interpret model projections and use them as an input to design policies, but also it enables model developers to envision potential avenues for improving models and better attune them in respect to their intended use. Model validation should thus be regarded as a critical step, and an integral part, of the iterative process of developing models for policy support.

### Supplementary Information

Below is the link to the electronic supplementary material.

Supplementary file1 (PDF 392 KB)

## Footnotes

1 The NUTS classification (Nomenclature of territorial units for statistics) is a hierarchical system for dividing up the territory of the EU for the purpose of collection, development and harmonisation of European regional statistics, as well as of socio-economic analyses of the regions. The main characteristics of NUTS 3 regions per country are summarised in Appendix A in the Supplementary Information.
