# Supplementary material for: Integrated Spatial Simulation of Population and Urban Land Use: a Pan-European Model Validation
Source: Appl Spat Anal Policy. 2023 Jun 1;16(4):1463–92. doi: 10.1007/s12061-023-09518-x (PMC10656325; doi:10.1007/s12061-023-09518-x)
Supplement: Supplementary file 1 — Supplementary file1 (PDF 392 KB) [file 12061_2023_9518_MOESM1_ESM.pdf]

Supplementary Information
Integrated spatial simulation of population and urban land use: a pan-European model validation

Appendix A. Characterisation of NUTS 3 regions in EU27+UK

Table A.1: Average size, share of country area and population, and number of NUTS 3 regions per country, according to the urban-rural regional typology defined by EUROSTAT (see https://ec.europa.eu/eurostat/statistics-explained/index.php?title=Archive:Urban-rural_typology for a definition of the typology)

	Predominantly urban				***Intermediate***				***Predominantly rural***				***Total***	
	Average size (km^2)	% country area	% country population	Nr. of regions	Average size (km^2)	% country area	% country population	Nr. of regions	Average size (km^2)	% country area	% country population	Nr. of regions	Average size (km^2)	Nr. of regions
**AT**	1490	7%	31%	4	2111	18%	28%	7	2631	75%	41%	24	2397	35
**BE**	488	21%	53%	13	706	45%	38%	19	831	34%	9%	12	675	44
**BG**	1342	1%	18%	1	4258	77%	69%	20	3498	22%	13%	7	3964	28
**CY**	-	-	-	-	9253	100%	100%	1	-	-	-	-	9253	1
**CZ**	5713	14%	24%	2	4803	49%	54%	8	7256	37%	21%	4	5634	14
**DE**	443	12%	43%	95	906	50%	41%	196	1254	39%	16%	110	892	401
**DK**	261	1%	22%	2	4071	47%	49%	5	5512	51%	29%	4	3902	11
**EE**	4338	10%	45%	1	4019	9%	11%	1	12326	82%	45%	3	9067	5
**GR**	938	6%	46%	8	2780	32%	24%	15	2845	63%	31%	29	2533	52
**ES**	8171	23%	62%	14	10685	60%	35%	28	10615	17%	4%	8	9970	50
**FI**	9568	3%	29%	1	8332	15%	31%	6	23238	82%	41%	12	17811	19
**FR**	3438	9%	35%	14	5913	31%	36%	29	6216	60%	30%	53	5719	96
**HR**	641	1%	19%	1	2906	36%	37%	7	2739	63%	44%	13	2695	21
**HU**	525	1%	17%	1	5140	72%	64%	13	4278	28%	19%	6	4651	20
**IE**	926	1%	27%	1	6889	10%	15%	1	10355	89%	58%	6	8743	8
**IT**	2143	21%	47%	29	2715	54%	43%	60	3669	26%	10%	21	2746	110
**LT**	9730	15%	28%	1	6669	72%	63%	7	4437	14%	9%	2	6528	10
**LU**	-	-	-	-	2595	100%	100%	1	-	-	-	-	2595	1
**LV**	304	0%	32%	1	12763	59%	46%	3	12996	40%	23%	2	10764	6
**MT**	158	100%	100%	2	-	-	-	-	-	-	-	-	158	2
**NL**	872	51%	74%	22	1026	47%	26%	17	749	2%	1%	1	934	40
**PL**	1011	5%	25%	14	4848	42%	39%	27	5215	54%	36%	32	4273	73
**PT**	2528	6%	46%	2	2244	13%	21%	5	4552	82%	33%	16	3874	23
**RO**	902	1%	11%	2	6244	31%	34%	12	5774	68%	55%	28	5676	42
**SE**	17966	8%	39%	2	21667	68%	52%	14	21632	24%	9%	5	21306	21
**SI**	-	-	-	-	1838	27%	41%	3	1640	73%	59%	9	1689	12
**SK**	2053	4%	11%	1	6102	50%	51%	4	7524	46%	38%	3	6129	8
**UK**	550	28%	74%	124	2764	42%	22%	37	4109	30%	4%	18	1365	179

Appendix B. Allocation module

B.1. Local suitability

The estimation of local population pressure and land-use suitability is based on an iterative procedure that starts with an empirical function explaining the attractiveness of a location for allocating residential population in a given time-step, as follows:

$$F_{i,t} = \beta_0 + \gamma Q_{i,t-1} + \delta W Q_{i,t-1} + \theta A_{i,t-1} + \beta_1 X_{1,i} + \cdots + \beta_s X_{s,i} \qquad \text{Eq. B1}$$

where:

$F_{i,t}$ is an empirical function representing the local attractiveness for residential functions, i.e. expected number of people in gridcell i in time-step t, as a function of a set of spatially-explicit explanatory variables;

β_0 is a constant;

$Q_{i,t-1}$ is the local population density (i.e. number of people) in gridcell i in the previous time-step *t-1*

$WQ_{i,t-1}$ is the prior average population density in the immediate neighbourhood of gridcell i, defined through a spatial weight matrix W (Queen's case), in the previous time-step *t-1*;

$A_{i,t-1}$ is the prior relative potential accessibility level in gridcell i in time-step *t-1*, calculated by dividing potential accessibility levels in gridcell i by the average values in the country of scope (see Jacobs-Crisioni et al., 2016);

γ, δ, θ are estimators of the effect of prior population, neighbouring population density and accessibility, respectively, on observed local population densities;

β_1 to β_s is a set of s parameters estimating the effect of the spatially-explicit explanatory variables $X_{1,i}$ to $X_{S,i}$ on observed local population densities;

$X_{1,i}$ to $X_{s,i}$ is a set of s local exogenous drivers assumed to be independent to prior local population densities, including slope, elevation, proximity to towns, proximity to leisure/touristic areas, proximity to roads, proximity to water, potential crop yields and zoning regulations (e.g. Natura 2000, nationally designated protected areas). For a detailed overview of the considered variables and data sources, see Appendix C. These variables do not fully cover, however, the whole range of factors that presumably govern the local residential attractiveness. A previous study has found substantial temporally-constant heterogeneity in municipal population density changes that appeared to be unaccounted for, when controlling for existing population densities, potential accessibility and explanatory spatial drivers (Jacobs-Crisioni and Koomen, 2017). We attempted to capture this heterogeneity through the estimation of fixed effects of population density change at municipal level for the relatively long period of 1961-2011, based on a separate panel data regression of municipal EU census data (Gløersen and Lüer, 2013). These fixed effects were then downscaled to the 100m resolution using inverse distance weighting interpolation. They were then included as an explanatory variable, in order to control for unobserved heterogeneity in population density among municipalities and capture established historical trends (implicitly assumed to be constant over time). Based on municipal population trends, this variable captures a different scale of change compared with variables that vary at a coarser grain (such as potential accessibility) or at a much finer grain (such as slope).

Eq. B1 was empirically fitted for all modelled countries separately, using Global Human Settlement Layer (GHSL) population data series (Florczyk et al., 2019) as a reference for observed population patterns. Concurrently, the total population to be (re-)allocated within a region was determined for every simulated time-step, as a function of the net population change in the region (positive or negative) and a fixed amount of people that is assumed to move residential location within the region in between time-steps, as follows:

$$K_{j,t} = \Delta Q_{j,t} + \sigma T Q_{j,t-1} \qquad \text{Eq. B2}$$

where:

$K_{j,t}$ is the total number of people to be (re-)allocated within a region in time-step t

$\Delta Q_{j,t}$ is the net population change in region j between time-step t and *t-1*

σ is a parameter indicating the amount of people changing residential location within region j each year (assumed to be 3.8%, i.e. the average intra-regional mobility according to the 2011 EU census).

T is the number of years between time-steps t and *t-1*

$Q_{j,t-1}$ is the prior number of people living in region j in time-step *t-1*

Finally, the total population to be (re-)allocated within a region and local attractiveness for residential functions are combined into a function of local population pressure, representing both the relative attractiveness of a location for allocating the expected changes in regional population and the potential local demand for residential functions:

$$Q_{i,t}^{pressure} = K_{j,t} \frac{F_{i,t}}{\sum_{i \in j} F_{i,t}} \qquad \text{Eq. B3}$$

where:
$Q_{i,t}^{pressure}$ is the local population pressure in gridcell i located in region j, in time-step t;
$K_{j,t}$ is the total population to be (re-)allocated within region j (see Eq. B2);
$F_{i,t}$ is the local attractiveness for residential functions of gridcell i located in region j, in time-step t (see Eq. B1).

Hence, local population pressure translates broader demographic changes at the regional level into relative changes in the local demand for providing residential functions (positive or negative), either by (de-)densifying existing urban areas (or even abandoning them), or by converting non-urban areas. This variable is then subsequently included as an endogenous driver in the estimation of urban land-use suitability. Country-specific land-use suitability functions were estimated separately for all simulated land-use types, through statistical analysis of observed land-use patterns. Since land-use change is conceptualised as a discrete phenomenon, we used the binomial logistic regression (BLR) model, a method commonly applied in land-change analysis to explain the occurrence of individual land-use types (Verburg et al., 2004). The BLR model transforms the dependent variable into a logit variable by estimating the odds of observing that land-use in a certain gridcell according to a set of spatially-explicit explanatory factors:

$$S_{i,k,t} = e^{(\alpha+\varphi X_{i,t}+\lambda W X_{i,t})}/(1 + e^{(\alpha+\varphi X_{i,t}+\lambda W X_{i,t})}) \qquad \text{Eq. B4}$$

where:
$S_{i,k,t}$ is the local land-use suitability, expressed as the probability of land-use type k occurring in gridcell i in year t, given a set of spatially-explicit explanatory factors assumed to determine the suitability of a location to support that land-use;
α is a constant;
φ is a vector of parameters estimating the effect of explanatory variables (i.e. spatially-explicit drivers) X on the local land-use suitability;
$X_{i,t}$ is the values of a set of spatially-explicit drivers in gridcell i, including the same set of exogenous drivers used in Eq. B1 and dynamic endogenous factors such as population pressure (Eq. B3) and accessibility (see Appendix C),;
$WX_{i,t}$ is the values of a set of local drivers X that are spatially-lagged in the immediate neighbourhood of gridcell i in year t, according to spatial weight matrix W (Queen's case);
λ is a vector of parameters estimating the effects of spatially-lagged local drivers WX on the local land-use suitability.

We used the same set of explanatory factors in the estimation of land-use suitability for urban residential land use and the other simulated land-use types. Coefficients were estimated through maximum likelihood estimation, indicating the direction and intensity of each spatially-explicit driver in explaining land-use suitability. Endogeneity issues invariably arise when explaining existing land-use patterns by variables that, themselves, depend on prior or current land use, likely causing biases in the estimated coefficients. For urban residential land use, this problem can be avoided by utilizing time series of built-up densities between 1990 and 2015 registered in the GHSL data series (Florczyk et al., 2019), as they entail fine resolution datasets that are consistently produced with identical remote sensing technology and interpretation models. The suitability functions for urban residential land use were thus fitted using land cover changes between 1990 and 2015 as observed in a subset of GHSL data series. Urban residential gridcells were assigned the value 1 in case they were not urban in 1990 and became urban by 2015, and 0 otherwise. For the other simulated land-use types, consistent time series are unavailable, or offering insufficient observations, for reliably fitting land-use suitability functions based on land-use changes (Lavalle et al., 2016). Hence, the suitability functions for land-use types other than urban residential (i.e. industry and commerce, agriculture, forestry) were fitted using CLC 2012 (Büttner et al., 2014) to represent the presence of land cover.

B.2. Local utility

Land-use activities require investments with a long-term time horizon (Diogo et al., 2015; Koomen et al., 2015). Local land-use utility is hereby computed as the expected net present value (NPV) of a given land use at a particular location, while taking into account its specific land-use suitability, expected revenues and costs. NPV is a standard method used in capital budgeting to appraise long-term investments, by measuring discounted time series of expected cash inflows and outflows, while taking into account the time value of money. To be regarded as economically attractive, a land-use investment should have a strictly non-negative NPV, after accounting for the expected annual costs and gross revenues associated with that particular land use within a given time-horizon, and their respective initial conversion costs. Land-use utility is thus computed as follows:

$$U_{i,k} = NPV_{i,k} = -I_{i,k} + \sum_{t=1}^{T_k} \frac{R_{i,k,t} - C_{k,t}}{(1+r_k)^t} \qquad \text{Eq. B5}$$

where:

$U_{i,k}$ is the land-use utility of a land-use type *k* in gridcell *i*

$NPV_{i,k}$ is the net present value of converting/maintaining the land in gridcell *i* to land-use type *k*

$I_{i,k}$ are the initial conversion costs in gridcell *i* that are incurred through the conversion of land to that land-use type *k* (in €/ha). Initial costs include the required investments on, for example, new buildings, facilities and machinery, and eventual land clearing costs, as the existing physical makeup of a location may call for specific demolition operations or vegetation clearing. In addition, empirical studies demonstrated that land systems appear to show inertia to land-use change partly as a result of the magnitude of previous investments on land and aversion to change activities under uncertainty (e.g. Regan et al., 2015; Schatzki, 2003). Hence, in the quantification of the initial conversion costs, we also account for eventual sunk costs (i.e. the investments on land and capital assets that may have already been made in the past for converting land into the existing land use) and opportunity costs (i.e. the current net benefits that would be foregone in case land is converted). Initial costs thus depend to a large extent on the prior existing land use in a particular location. They are considered to be 0 if the gridcell is already under the land use for which NPV is being estimated. Land-use types requiring large investments or yielding high net revenues are typically given larger sunk costs and opportunity costs, respectively. This implies that existing land use may not be converted easily, even when an alternative land use would potentially yield higher net revenues.

$R_{i,k,t}$ is the annual gross revenues that can be obtained in gridcell *i* in year *t* from land-use type *k* (in €/ha/year), including revenues obtained from e.g. rental income, selling crops, subsidies, monetary valuation of ecosystem services, etc.;

$C_{k,t}$ is the annual costs in year *t* associated with that land-use type *k* (in €/ha/year), e.g. maintenance costs, agricultural field operations and inputs, taxes, depreciation, etc.;

T_k is the investment time-horizon of land-use type *k* (in years);

r_k is the discount rate associated with land-use type *k*.

The annual gross revenues are measured through a proxy calculated by rescaling assumed maximum annual revenues of a given land-use type by the respective local land-use suitability function, as follows:

$$R_{i,k,t} = S_{i,k,t} * \max R_{k,t} \qquad \text{Eq. B6}$$

where:

$R_{i,k,t}$ is the annual gross revenues that can be obtained from land-use type *k* in gridcell *i* in year *t* (in €/ha/year);

$S_{i,k,t}$ is the local land-use suitability of land-use type *k* in gridcell *i* in year *t*, as estimated in Eq. B4 above;

$\max R_{k,t}$ are the assumed maximum annual revenues (in €/ha), i.e. the gross revenues obtained from land-use type *k* when the local land-use suitability is optimal (i.e. $S_{i,k,t}$ = 100%).

Eq. B6 thus aims to capture that land-use revenues are highly location-dependent, for example, due to differences in local land-use suitability (e.g. house prices are often determined by the existence of local amenities, crop yields depend on a combination of local biophysical characteristics) and location-specific costs not captured by the base annual costs $C_{k,t}$ (e.g. transportation costs of commodities). The calculated NPVs are not presumed to be exact estimations of land-use utility, but rather to provide a local measure of the relative competitiveness among different land use alternatives in each location. Initial costs, maximum revenues, annual costs, discount rates and time-horizon were specified for the different simulated land-uses through a combination of literature review, available databases, official statistics and modelling results from specialised models (see Appendix D for a specification of these variables for the Netherlands, as an illustrative example).

B.3. Simulation of land-use and population patterns

The simulated land-use types are dynamically allocated so that the overall utility derived from land use within a region is maximised in every time-step, taking into account previous land-use and population patterns, local utility of alternative land-use activities and their expected land demand. For that purpose, we implement the discrete version of the spatial allocation algorithm proposed in Hilferink and Rietveld (1999; see Koomen et al., 2011 for a detailed description of the continuous and discrete models), which mimics iterative bidding processes in the land market. The algorithm is doubly constrained by, on the one hand, the regional land demand and, on the other hand, the regional land supply (i.e. the amount of land available to accommodate these activities), as follows:

$$M_{i,k} = a_k \,.\, b_i \,.\, e^{\beta . U_{i,k}} \text{, for all simulated land-use types } k \text{ and gridcells } i \qquad \text{Eq. B7}$$

with a_k and b_i being specified so that the constraints below (i.e. Eqs. B8, B9 and B10) are satisfied:

$$\{M_{i,k}|0, G_i\}$$ Eq. B8

$$\sum_{k \in N} M_{i,k} = G_i$$ Eq. B9

$$\sum_{i \in j} M_{i,k} = D_{k,j} \text{ , for all simulated land-use types } k$$ Eq. B10

where:

$M_{i,k}$ is the area of land-use type k allocated in gridcell i, which according to the constraint specified in Eq. B8 can only be 0 (i.e. no land is allocated to land-use type k) or G_i (i.e. the whole gridcell size area, in this case 1 hectare, is allocated to land-use type k);

$U_{i,k}$ is the land-use utility of land-use type k in gridcell i (i.e. the NPV of land-use type k in that location, as specified in Eq. B5);

β is a parameter to adjust the model sensitivity (typically, 1 as default value);

a_k is the demand balancing factor, which ensures that the total amount of allocated land for the simulated land-use type k equals its respective regional land demand $D_{k,j}$;

b_i is the supply balancing factor, which ensures that the total amount of allocated land in gridcell i does not exceed its size G_i;

G_i is the area of gridcell i (i.e. 1 hectare);

N is the number of simulated land-use types;

$D_{k,j}$ is the total aggregated land demand for the simulated land-use type k in region j, as specified by the regional demand module (in hectares).

Solving the model entails finding the sets of values for all a_k and b_i that are equal to:

$$a_k = \frac{D_{k,j}}{\sum_{i \in j} b_i \,.\, e^{\beta.U_{i,k}}}$$ Eq. B11

$$b_i = \frac{G_i}{\sum_{k=1}^{n} a_k \,.\, e^{\beta.U_{i,k}}}$$ Eq. B12

The appropriate values for a_k and b_i are found through an iterative approach mimicking a bidding process between competing land uses, in which each land-use attempts to get its total demand satisfied, but may be outbidded in a given location by another land use that derives higher utility from land. For a detailed description of the iterative bidding process, we refer to Hilferink and Rietveld (1999).

After the spatial allocation of land use (including urban residential land use), population is then redistributed over the region. The method to do so is similar to the way population pressure is computed (Eq. B3). However, a few additional elements are introduced to ensure that the observed inertia of the population patterns is maintained. Most importantly, the empirical function explaining local attractiveness for allocating population (i.e. Eq. B1) is refitted on specific subsets of the calibration data that, through their selection mechanism, include the effects of allocated urban land use, as follows:

$$F'_{i,t} = F_{i,t}\, residential + F_{i,t}\, residential\ remote + F_{i,t}\, non\ residential$$ Eq. B13

$F'_{i,t}$ is an empirical function representing the local attractiveness for allocating population in gridcell i in time-step t, given a set of spatially-explicit explanatory factors, and according to subsets of the calibration data;

$F_{i,t}\ residential$ is an empirical function similar to Eq. B1, refitted on a set of the calibration data where land use is classified as urban residential and travel time to the closest major urban cluster is up to 45 minutes. Major urban clusters are defined consistently across Europe as patches of contiguous urban residential land use that together have at least 30,000 inhabitants, and a minimum population of 5,000;

$F_{i,t}\ residential\ remote$ is an empirical function similar to Eq. B1, refitted on a set of the calibration data where land use is classified as urban residential and travel time to the closest major urban cluster is longer than 45 minutes;

$F_{i,t}\ non\ residential$ is an empirical function similar to Eq. B1, refitted on a set of the calibration data where land use is not predominantly urban residential, but people were living there in the previous time-step *t-1*.

The three subsets of $F_{i,t}$ are mutually exclusive per gridcell, so that the result is always zero for two of the subset functions. Population changes are thus only possible if a grid cell is urban residential, has just become urban

residential, or is not urban residential but already had population at the reference starting point. The inclusion of different subset functions is meant to account for distinct location preferences in different segments of the real estate market. Population distributions are then iteratively allocated, as a function of previous local population density and the relative attractiveness of a location for allocating the expected changes in regional population (for a detailed description of the allocation algorithm, see Jacobs-Crisioni et al., 2017):

$$Q_{i,t} = (1 - \sigma T)Q_{i,t-1} + K_{j,t}\frac{F'_{i,t}}{\sum_{i' \in j} F'_{i,t}} \;\; , \;\; Q_{i,t} > 0 \qquad \text{Eq. B14}$$

where:
$Q_{i,t}$ is the number of people allocated in gridcell *i* in time-step *t*
$Q_{i,t-1}$ is the prior number of people in gridcell *i* in time-step *t-1*
$K_{j,t}$ is the total population to be (re-)allocated within a region (see Eq. B2)
σ is the parameter indicating the amount of people changing residential location within region *j* each year
T is the number of years between time-steps *t* and *t-1*
$F'_{i,t}$ is the local attractiveness to allocate population in gridcell *i* located in region *j*, as explained by an empirical function given a set of spatially-explicit explanatory factors, and according to subsets of calibration data (see Eq. B13)

Appendix C. Spatial explanatory factors

Table C.1: Variables used as explanatory factors in Eqs. B1, B4 and B13

Internal code	Description	Eq. B1	Eq. B4	Eq. B13
ln_yieldmaize	Natural log of estimated maize yield[1]	N	Y	N
ln_yieldbarley	Natural log of estimated barley yield[1]	N	Y	N
ln_yieldsugbeet	Natural log of estimated sugar beets yield[1]	N	Y	N
ln_yieldsunflow	Natural log of estimated sunflower yield[1]	N	Y	N
ln_yieldwheat	Natural log of estimated wheat yield[1]	N	Y	N
ln_yieldrapeseed	Natural log of estimated rapeseed yield[1]	N	Y	N
TownAccess	Travel time to closest town[2]	Y	Y	Y
RelDomAccess	Relative domestic potential accessibility[2]	Y	Y	Y
RelPotAccess	Relative total potential accessibility[2]	N	Y	N
pop_fe_1	Spatially interpolated municipality fixed effects[2]	Y	Y	Y
Tourism_attr	Number of tourist attractions in grid cell[3]	N	Y	N
Tourism_hotel	Number of hotels in grid cell[3]	N	Y	N
Tourism_rest	Number of restaurants in grid cell[3]	N	Y	N
Tourism_vacrent	Number of vacation rentals in grid cell[3]	N	Y	N
Tourism_attr_km10	Euclidean distance to closest tourist attraction[3]	N	Y	N
Tourism_rest_km10	Euclidean distance to closest restaurant[3]	N	Y	N
ln_roads	Natural log of distance to main roads[4]	Y	Y	Y
ln_water	Natural log of distance to main water bodies	Y	Y	Y
slope100_cont	Slope angle[5]	Y	Y	Y
Elevation_100	Elevation[5]	N	Y	N
natura2000_v2011	Dichotomous indication in a Natura 2000 area[6]	Y	Y	Y
NDA	Dichotomous indication in a nationally designated natural protection area[6]	N	Y	N
PriorPopulation	Number of inhabitants in the prior time-step	Y	N	Y
PopulationPressure	Estimated population pressure (*F* in Eq. B1)	N	Y	N
UrbanLandUse	Indicates whether a grid cell is urban, post discrete land-use allocation (from Eq. B12)	N	N	Y

Data sources:
[1] yield maps from the BIOMA project (Donatelli et al., 2015);
[2] accessibility and municipality fixed effect estimates, which combine CORINE derivates with TRANS-TOOLS road networks using methods from Jacobs-Crisioni and Koomen (2017);
[3] based on tourism-specific data obtained from the online TripAdvisor database;
[4] based on roads of at least class 3 according to the TomTom roads network database.
[5] COPERNICUS EU digital elevation model.
[6] inventories of Natura 2000 and nationally designated areas, as provided by EEA.

Appendix D. Economic drivers

Table D.1: Example of valuation of land-use utility in the Netherlands, and respective data sources

Land-use type	Clearing costs (EUR/m^2)	Investment costs (EUR/m^2)	Annual costs (EUR/m^2)	Maximum annual revenues (EUR/m^2)	Discount rate	Time horizon (years)
Urban residential	57.00 [1]	1126.00 [1]	20.47 [2]	464.00 [3]	10.0% [4]	10 [4]
Industrial	25.00 [1]	1065.00 [5]	19.36 [2]	556.00 [6]	10.0% [4]	10 [4]
Arable crops	0.04 [7]	0.73 [7]	0.36 [7]	0.71 [7]	5.5% [7]	20 [7]
Livestock production	0.04 [7]	1.20 [7]	0.25 [7]	0.70 [7]	5.5% [7]	20 [7]
Permanent crops	0.81 [7]	3.59 [7]	0.69 [7]	1.78 [7]	5.5% [7]	20 [7]
Forest	1.61 [8]	1.38 [9]	0.03 [9]	0.67 [10]	3.0% [10]	50 [9]
(Semi-)natural vegetation	0.27 [8]	-	-	0.52 [10]	2.0% [10]	100 [10]

Data sources:
[1] Claassens et al. (2019)
[2] Assuming 2% annual depreciation of buildings (EY, 2010)
[3] GPG (2017)
[4] Cárdenas (2012)
[5] SA (2018)
[6] David and Can (2014)
[7] Diogo et al. (2015)
[8] CH (2017)
[9] Teeuwen et al. (2020)
[10] Horlings et al. (2020); annual revenues of (semi-)natural vegetation were assumed to be the average of the monetary valuation of the ecosystem services provided by (semi-)natural grassland, heathland and inland dunes

References

Büttner, G., Soukup, T., Kosztra, B., 2014. CLC2012 Addendum to CLC2006 Technical Guidelines. European Environment Agency. Copenhagen, Denmark.

Cárdenas, J.S., 2012. The discount rate for income properties. Harvard Deusto Bus. Res. 1, 147–157. https://doi.org/10.3926/HDBR.28

CH, 2017. Land clearing costs. CostHelper.

Claassens, J., Hilferink, M., Rijken, B.C., Koomen, E., 2019. The financial feasibility of residential development options in the existing city. Spinlab Research Memorandum; Vol. SL-15. Vrije Universiteit Amsterdam, Amsterdam.

David, B., Can, E., 2014. Office space across the world. Cushman & Wakefield, European Research Group.

Diogo, V., Koomen, E., Kuhlman, T., 2015. An economic theory-based explanatory model of agricultural land-use patterns: The Netherlands as a case study. Agric. Syst. 139, 1–16. https://doi.org/10.1016/j.agsy.2015.06.002

Donatelli, M., Srivastava, A.K., Duveiller, G., Niemeyer, S., Fumagalli, D., 2015. Climate change impact and potential adaptation strategies under alternate realizations of climate scenarios for three major crops in Europe. Environ. Res. Lett. 10. https://doi.org/10.1088/1748-9326/10/7/075005

EY, 2010. Depreciation on buildings: Financial reporting and tax considerations. Ernst & Young.

Florczyk, A.J., Corbane, C., Ehrlich, D., Freire, S., Kemper, T., Maffenini, L., Melchiorri, M., Pesaresi, M., Politis, P., Schiavina, M., Sabo, F., Zanchetta, L., 2019. GHSL Data Package. Publications Office of the European Union, Luxembourg.

Gløersen, E., Lüer, C., 2013. Population data collection for European local administrative units from 1960 onwards. European Commission. Publications Office of the European Union. Luxembourg.

GPG, 2017. Global Property Guide. URL http://www.globalpropertyguide.com/

Hilferink, M., Rietveld, P., 1999. LAND USE SCANNER: An integrated GIS based model for long term projections of land use in urban and rural areas. J. Geogr. Syst. 177, 155–177.

Horlings, E., Schenau, S., Hein, L., Lof, M., de Jongh, L., Polder, M., 2020. Experimental Monetary Valuation of Ecosystem Services and Assets in the Netherlands. Statistics Netherlands, Wageningen University.

Jacobs-Crisioni, C., Batista, F., Lavalle, C., Baranzelli, C., Barbosa, A., Castillo, C.P., 2016. Accessibility and territorial cohesion in a case of transport infrastructure improvements with changing population distributions. Eur. Transp. Res. Rev. https://doi.org/10.1007/s12544-016-0197-5

Jacobs-Crisioni, C., Diogo, V., Perpiña Castillo, C., Baranzelli, C., Batista e Silva, F., Rosina, K., Kavalov, B., Lavalle, C., 2017. The LUISA Territorial Reference Scenario: A technical description. Union, JRC Technical Report. Joint Research Centre of the European Commission. Publications office of the European. Luxembourg. https://doi.org/10.2760/902121

Jacobs-Crisioni, C., Koomen, E., 2017. Population growth, accessibility, spillovers and persistent borders: Historical growth in West- European municipalities. J. Transp. Geogr. 62, 80–91. https://doi.org/10.1016/j.jtrangeo.2017.05.008

Koomen, E., Diogo, V., Dekkers, J., Rietveld, P., 2015. A utility-based suitability framework for integrated local-scale land-use modelling. Comput. Environ. Urban Syst. 50, 1–14. https://doi.org/10.1016/J.COMPENVURBSYS.2014.10.002

Koomen, Eric, Hilferink, Maarten, Borsboom-van Beurden, Judith, 2011. Introducing Land Use Scanner, in: Koomen, E., Hilferink, M., Borsboom-van Beurden, J. (Eds.), Land-Use Modelling in Planning Practice. Springer, Dordrecht, pp. 3–21. https://doi.org/10.1007/978-94-007-1822-7_1

Lavalle, C., Batista e Silva, F., Baranzelli, C., Jacobs-Crisioni, C., Vandecasteele, I., Barbosa, A.L., Maes, J., Zulian, G., Castillo, C.P., Barranco, R., Vallecillo, S., 2016. Chapter 24: Land Use and Scenario Modeling

for Integrated Sustainability Assessment, in: Feranec, J., Soukup, T., Hazeu, G., Jaffrain, G. (Eds.), European Landscape Dynamics: CORINE Land Cover Data . CRC Press, Boca Raton, pp. 237–262. https://doi.org/10.1201/9781315372860-25

Regan, C.M., Bryan, B.A., Connor, J.D., Meyer, W.S., Ostendorf, B., Zhu, Z., Bao, C., 2015. Real options analysis for land use management : Methods , application , and implications for policy. J. Environ. Manage. 161, 144–152. https://doi.org/10.1016/j.jenvman.2015.07.004

SA, 2018. Average warehouse building cost per square meter in selected European cities in 2018. Stat. Accounts. URL https://www.statista.com/statistics/989710/warehouse-building-costs-european-cities/

Schatzki, T., 2003. Options, uncertainty and sunk costs: An empirical analysis of land use change. J. Environ. Econ. Manage. 46, 86–105. https://doi.org/10.1016/S0095-0696(02)00030-X

Teeuwen, S., Reichgelt, A., Oldenburger, J., 2020. Kostenindicatie aanleg nieuw bos en landschapselementen: Rekenhulpmiddel voor het ramen van de kosten voor aanplant van verschillende beplantingstypen. Stichting Probos, Vereniging van Bos- en Natuurterreineigenaren (VBNE).

Verburg, P.H., Dijst, M.J., Schot, P., 2004. Determinants of land-use change patterns in the Netherlands. Environ. Plann. B 31. https://doi.org/10.1068/b307
